# Supplementary material for: Reconstruction of zinc-metal battery solvation structures operating from −50 ~ +100 °C
Source: Nat Commun. 2024 Jul 24;15:6249. doi: 10.1038/s41467-024-50219-x (PMC11269709; doi:10.1038/s41467-024-50219-x)
Supplement: Supplementary file 1 — Supplementary Information [file 41467_2024_50219_MOESM1_ESM.pdf]

Supplementary Information

Reconstruction of Zinc-metal battery solvation structures operating from -50~+100 °C

Lingbo Yao[1,2], Jiahe Liu[1,2], Feifan Zhang[1,2], Bo Wen[1,2], Xiaowei Chi[1*], Yu Liu[1*]

[1]Shanghai Institute of Ceramics, Chinese Academy of Sciences

Shanghai 200050, China

[2]University of Chinese Academy of Sciences

Beijing 100049, China

*Email: xwchi@mail.sic.ac.cn; yuliu@mail.sic.ac.cn

Contents

Contents 2

Supplementary Figures 8

Supplementary Fig. 1 | The reaction between boric acid and glycerol. **a,** The reversible esterification reaction between boric acid and glycerol. **b,** pH changes of the boric acid solution before and after adding glycerol..8

Supplementary Fig. 2 | The precursor solution before UV cross link using different electrolytes. **a,** $Zn(BF_4)_2$. **b,** $ZnCl_2$, and **c,** $Zn(ClO_4)_2$. 9

Supplementary Fig. 3 | Thermal properties of different hydrogel electrolytes. **a,** TG profiles of ZCP, ZGP, and ZGBCP electrolytes. **b,** DSC profile of ZGBCP electrolyte. **c,** Flaming experiment of ZGBCP electrolyte.....................................10

Supplementary Fig. 4 | Schematic diagram of ZGBCP hydrogel adhering to various electrodes and current collector materials. 11

Supplementary Fig. 5 | Shear adhesion tests of the ZGBCP, ZGP, and ZCP electrolytes adhering to the Zn anodes..12

Supplementary Fig. 6 | Optical microscopic images of the interface between different hydrogels **a,** ZGP hydrogel, **b,** ZCP hydrogel, and **c,** ZGBCP hydrogel adhesion to Zn electrode. 13

Supplementary Fig. 7 | **a,** Molecule models used in the MD simulations. **b,** Binding energies of Zn^{2+} and BF_4^- with two H_2O molecules. 14

Supplementary Fig. 8 | MD simulation of AE and ZGBCP electrolytes. **a,** MD snapshot of ZGBCP electrolyte. **b,** g(r) and n(r) of the Zn^{2+} in the AE electrolyte. **c,** Electrostatic interaction energy of the H sites in H_2O of $[Zn^{2+}BF_4^-(H_2O)_5]^+$ and $[Zn^{2+}BF_4^-(H_2O)_4(C_3H_8O_3)]^+$-PAM based on the B97-3c level DFT calculations. .15

Supplementary Fig. 9 | Distribution of the solvent separated ions pairs (SSIP) and contact ion pairs (CIP) in the **a,** AE, and **b,** ZGBCP electrolyte......................16

Supplementary Fig. 10 | Various cationic solvation structures obtained from MD simulations and corresponding solvation energy based on the B97-3c level DFT calculations. **a,** $[Zn^{2+}(BF_4^-)(H_2O)_5]^+$ and $[Zn^{2+}(H_2O)_6]^{2+}$ in the AE electrolyte, and **b,** $[Zn^{2+}(BF_4^-)(H_2O)_4(C_3H_8O_3)]^+$-PAM, $[Zn^{2+}(BF_4^-)(H_2O)_3(C_3H_8O_3)]^+$-PAM, $[Zn^{2+}(BF_4^-)_2(H_2O)_2(C_3H_8O_3)]$-CS, $[Zn^{2+}(BF_4^-)(H_2O)_4(C_3H_8O_3)]^+$, and $[Zn^{2+}(H_2O)_5(C_3H_8O_3)]^{2+}$ in the ZGBCP electrolyte..17

Supplementary Fig. 11 | The CIP-type solvation structure without polymer involved based on the B97-3c level DFT calculations. **a,** The ESP distribution, **b,** Electrostatic interaction energy of the H sites in H_2O of CIP-type cationic solvation structure $[Zn^{2+}BF_4^-(H_2O)_4(C_3H_8O_3)]^+$ and $[Zn^{2+}(BF_4^-)(H_2O)_5]^+$.............................18

Supplementary Fig. 12 | The SSIP-type solvation structure without polymer involved based on the B97-3c level DFT calculations. **a,** The ESP distribution, **b,** Electrostatic interaction energy of the H sites in H_2O of SSP-type cationic solvation structures $[Zn^{2+}(H_2O)_5(C_3H_8O_3)]^{2+}$ and $[Zn^{2+}(H_2O)_6]^{2+}$. 19

Supplementary Fig. 13 | The cationic solvation structure tuned by the CS **a,** Binding energy of PAM-Zn^{2+} and CS-Zn^{2+} based on the B3LYP/6-311g++(d,p) level DFT calculations. **b,** Binding energy of PAM-BF_4^- and CS-BF_4^- based on the B3LYP/6-311g++(d,p) level DFT calculations. **c,** g(r) and n(r) of overall Zn^{2+} from CS. **d,** n(r) of the Zn^{2+} coordinated by CS nearby. **e,** The ESP distribution, and **f,** Electrostatic interaction energy of the H sites in H_2O of cationic solvation structures $[Zn^{2+}(BF_4^-)_2(H_2O)_2(C_3H_8O_3)]$-CS based on the B97-3c level DFT calculations.....20

Supplementary Fig. 14 | The MD simulations based on the pentamers models. **a,** Molecular models of PAM and CS pentamers used in the MD simulations. **b,** g(r) and n(r) of Zn^{2+}. **c,** g(r) of F in the ZGBCP_5mer system. **d,** n(r) of F-H (H_2O) in the ZGBCP_3mer and ZGBCP_5mer systems. 21

Supplementary Fig. 15 | Laplacian distribution diagrams and mayer bond order for BF_4^- interactions with **a,** H_2O, **b,** $C_3H_8O_3$, and **c,** H_3BO_3 based on the B3LYP/6-311++g(d,p) level DFT calculations. 22

Supplementary Fig. 16 | Distribution and structures of free and coordinated BF_4^-. **a,** Distribution of the free and coordinated BF_4^- in the AE. **b,** The solvation structure of free BF_4^- in the AE. **c,** Distribution of the free and coordinated BF_4^- in the ZGBCP electrolyte. **d,** The g(r) of BF_4^- in the ZGBCP electrolyte. **e,** 23

Supplementary Fig. 17 | **a,** Energy level, and **b,** optimized models of different potential solvation structures of free anions denoted as $BF_4^-(H_2O)_a(C_3H_8O_3)_b(H_3BO_3)_c(PAM)_d(CS)_e$ based on the B97-3c level DFT calculations. 24

Supplementary Fig. 18 | The temperature-dependent EIS tests of the Zn||Zn symmetric cells using **a,** ZGBCP, **b,** ZCP, **c,** ZGP, and **d,** AE electrolytes.............25

Supplementary Fig. 19 | Ionic transport property calculated by the MD simulation under different temperatures. **a,** MSD of Zn^{2+} obtained from MD simulations under different temperatures. **b,** diffusion coefficients and **c,** conductivities of ZGBCP electrolytes based on simulation and experiments under different temperatures. 26

Supplementary Fig. 20 | The analysis of acceleration effect of desolvation tuned by the glycerol. **a,** Schematic diagram of rigid scan of Zn^{2+} rotating around glycerol molecule at a Zn-O-C-C dihedral angle θ. **b,** Binding energy between the Zn^{2+} and glycerol based on the rigid scan with B3LYP/6-311++g(d,p) level DFT calculation. **c,** Raman spectra of the pure glycerol, 1,2-dipropanol, and 1,3-dipropanol. **d,** Chronoamperometry tests. **e,** Voltage-time profiles of Zn||Zn symmetrical batteries using ZGP, Z12PP, and Z13PP electrolytes. **f,** Desolvation energy barrier $\triangle E_{desol}$ of $[Zn^{2+}(BF_4^-)(H_2O)_4(C_3H_8O_3)]^+$-PAM and $[Zn^{2+}(BF_4^-)(H_2O)_5]^+$ obtained from the DFT calculation based on the B97-3c level DFT calculations.......................................27

Supplementary Fig. 21 | The spectral characterizations of the different electrolytes. **a,** ATR-FT-IR spectra, and corresponding **b,** Local enlarge, and **c,** First order differentiation. 29

Supplementary Fig. 22 | ^{19}F NMR spectra of AE electrolyte added

with/without boric acid. 30

Supplementary Fig. 23 | **a,** The full spectra and **b,** XPS F 1*s* spectra of ZGBCP, ZGP, and ZCP electrolytes...31

Supplementary Fig. 24 | The in-situ optical observation of the Zn plating and stripping reaction at the interface between Zn and electrolytes (top: ZGBCP electrolyte; bottom: AE electrolyte)..32

Supplementary Fig. 25 | The electrochemical analysis of the reaction kinetics of the Zn anodes using different electrolytes. **a,** Chronoamperometry curves under polarization potential of −20 mV. **b,** Differential capacitance curves. **c,** DRT analysis of the Zn||Zn symmetric cells. **d,** Tafel tests for the reaction kinetics of Zn anodes using different electrolytes. 33

Supplementary Fig. 26 | Optical profile reconstruction of the anodes using **a,** ZGBCP and **b,** AE electrolyte and **c,** roughness tests of the cycled anodes using different electrolytes. 34

Supplementary Fig. 27 | SEM images of the Zn anodes after 50 cycles using **a-b,** ZGBCP. **c,** AE electrolyte. 35

Supplementary Fig. 28 | SEM images corresponding EDS mappings of the cross-sections of Zn anodes using **a,** ZGBCP and **b,** AE electrolytes. 36

Supplementary Fig. 29 | TEM images of the cross-section of the Zn anodes using ZGBCP electrolytes after 10 cycles..37

Supplementary Fig. 30 | The XPS survey and corresponding C 1*s* spectra of Zn anodes at different depths using **a,** ZGBCP, and **b,** AE electrolyte...................38

Supplementary Fig. 31 | XPS F 1*s* spectra of the cycled Zn anodes surface in different depth using **a,** ZGBCP, and **b,** AE electrolyte...39

Supplementary Fig. 32 | Stability and kinetics of stripping/plating reaction characterized by Tafel tests using different electrolytes under **a,** -40 °C, and **b,** 50 °C. 40

Supplementary Fig. 33 | The characterizations of CPZ electrodes. **a,** SEM image.

b, DRT analysis and **c,** rate performance of CPZ||CPZ symmetric cell using the ZGBCP electrolyte. 41

Supplementary Fig. 34 | The rate performance of the Zn||Zn symmetric batteries using **a,** ZGBCP, **b,** ZCP, **c,** ZGP, and **d,** AE electrolytes under -40 °C....42

Supplementary Fig. 35 | Kinetics analysis and comparison of the full batteries using PANI cathodes. **a,** GCD profiles of the full batteries using AE electrolyte. **b,** Average CE and average EE under different current densities. **c,** Ragone plots of the full batteries of this work compared with reported works using PANI cathodes[2-7]. **d,** GITT tests using the AE, and ZGBCP electrolytes. 43

Supplementary Fig. 36 | Rate and cycling performance of the Zn|ZGBCP|I_2 full cell. **a,** GCD profiles under different current densities. **b,** cycling performance at 15 A g^{-1}. 44

Supplementary Fig. 37 | CV profiles of the Zn||PANI full batteries using **a-b,** ZGP, and **c-d,** ZCP electrolytes. 45

Supplementary Fig. 38 | Reaction kinetics analysis of the Zn|ZGBCP|PANI full cells. **a,** Variable sweep rate (v, 1-10 mV s^{-1}) CV curves. **b,** Proportion of pseudocapacitance contribution using ZGBCP electrolyte at different scanning speeds from 0.2 to 1 mV s^{-1}. 46

Supplementary Fig. 39 | Energy level of different cationic solvation structures in the AE (right) and ZGBCP (left) electrolytes, respectively based on the B97-3c level DFT calculations. **a,** $[Zn^{2+}(BF_4^-)(H_2O)_4(C_3H_8O_3)]^+$ and $[Zn^{2+}(BF_4^-)(H_2O)_5]^+$, **b,** $[Zn^{2+}(H_2O)_5(C_3H_8O_3)]^{2+}$ and $[Zn^{2+}(H_2O)_6]^{2+}$, **c,** $[Zn^{2+}(BF_4^-)_2(H_2O)_2(C_3H_8O_3)]$-CS and $[Zn^{2+}(BF_4^-)(H_2O)_5]^+$. **d**, The LUMO and HOMO orbitals of the cationic solvation structures. 47

Supplementary Fig. 40 | **a,** Energy level and corresponding **b,** orbital structures of different solvation structures of free anions denoted as $BF_4^-(H_2O)_a(C_3H_8O_3)_b(H_3BO_3)_c(PAM)_d(CS)_e$ based on the B97-3c level DFT calculations. 48

Supplementary Fig. 41 | Spectral characterization and mechanism analysis of PANI electrodes using ZGBCP and ZCP electrolyte. **a,** XPS N 1*s* spectra after 1000 cycles under room temperature. **b,** Evolution of the UV-Vis spectra along with the cycling under (50 °C). **c,** Raman spectra of the PANI cathode using ZGBCP electrolyte after 1000 cycles under 50 °C. 50

Supplementary Fig. 42 | Low temperature electrochemistry performance of Zn|ZGBCP|PANI full batteries. **a,** Cycling performance under -40 °C. **b,** Rate performance under -50 °C. 51

Supplementary Fig. 43 | Electrochemical performance of full batteries using AE and ZGBCP electrolytes under high temperatures. **a,** CV curves at 50 °C. **b,** GCD profiles at 50 °C. **c,** Cycling performance at 80 °C 52

Supplementary Fig. 44 | The electrochemical performance and demonstration for powering electronic devices of Zn||PANI pouch-cells using ZGBCP electrolyte. **a,** Cycling performance. **b,** Voltage retention at 50 °C. **c,** Demonstration of wearable devices powered by the pouch-cell in series. 53

References 57

Supplementary Figures

a

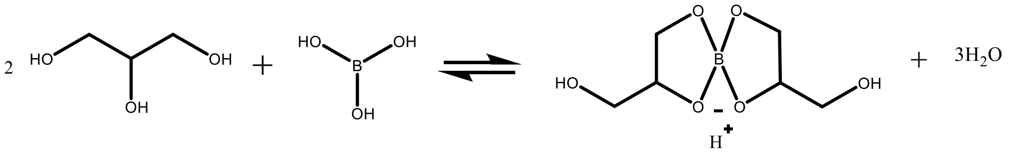

b

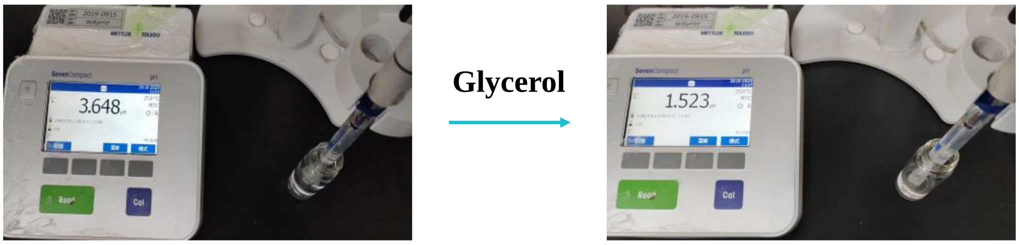

Supplementary Fig. 1 | The reaction between boric acid and glycerol. **a,** The reversible esterification reaction between boric acid and glycerol. **b,** pH changes of the boric acid solution before and after adding glycerol.

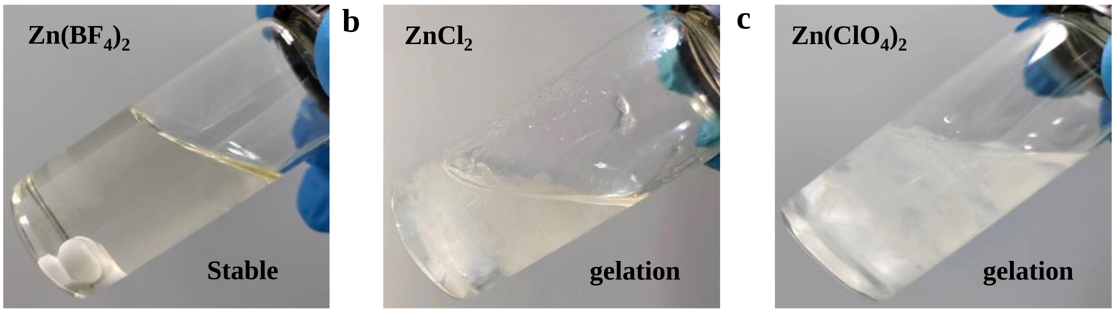

Supplementary Fig. 2 | The precursor solution before UV cross link using different electrolytes. **a,** $Zn(BF_4)_2$. **b,** $ZnCl_2$, and **c,** $Zn(ClO_4)_2$.

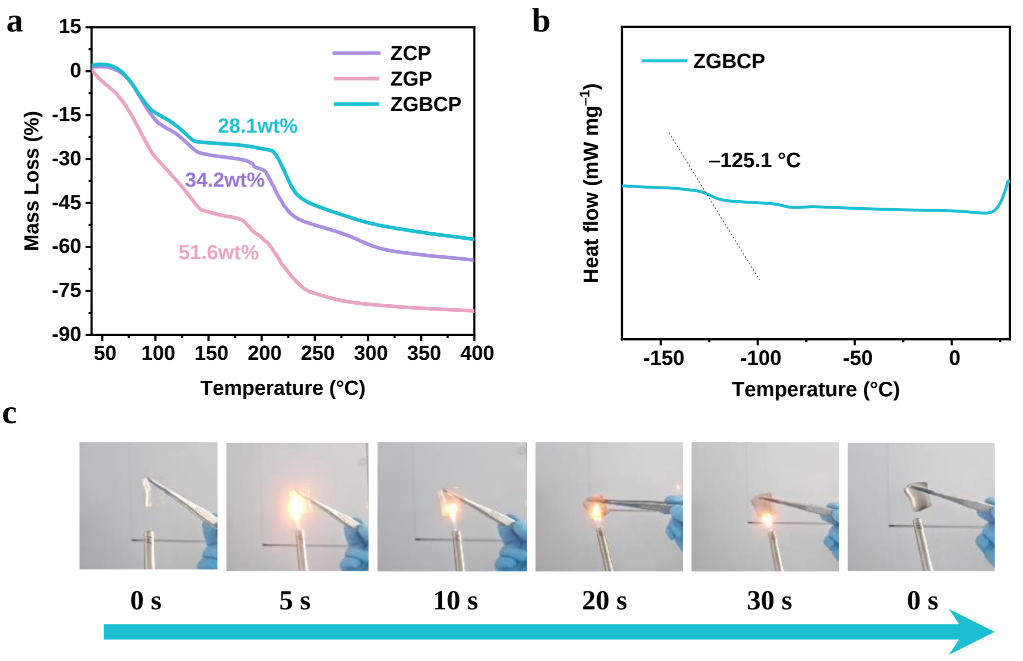

Supplementary Fig. 3 | Thermal properties of different hydrogel electrolytes. **a,** TG profiles of ZCP, ZGP, and ZGBCP electrolytes. **b,** DSC profile of ZGBCP electrolyte. **c,** Flaming experiment of ZGBCP electrolyte.

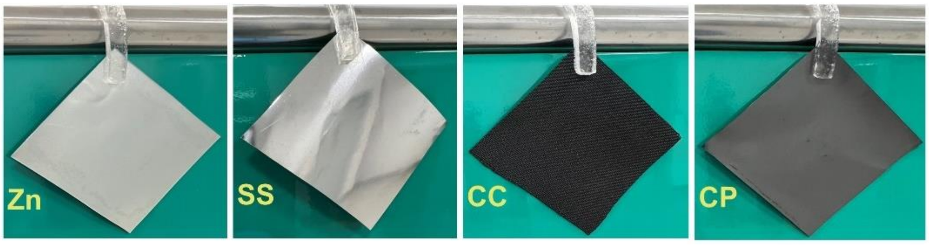

Supplementary Fig. 4 | Schematic diagram of ZGBCP hydrogel adhering to various electrodes and current collector materials.

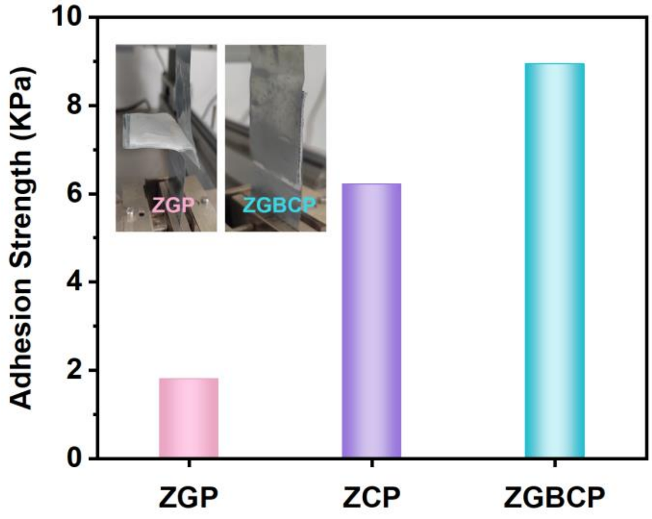

Supplementary Fig. 5 | Shear adhesion tests of the ZGBCP, ZGP, and ZCP electrolytes adhering to the Zn anodes.

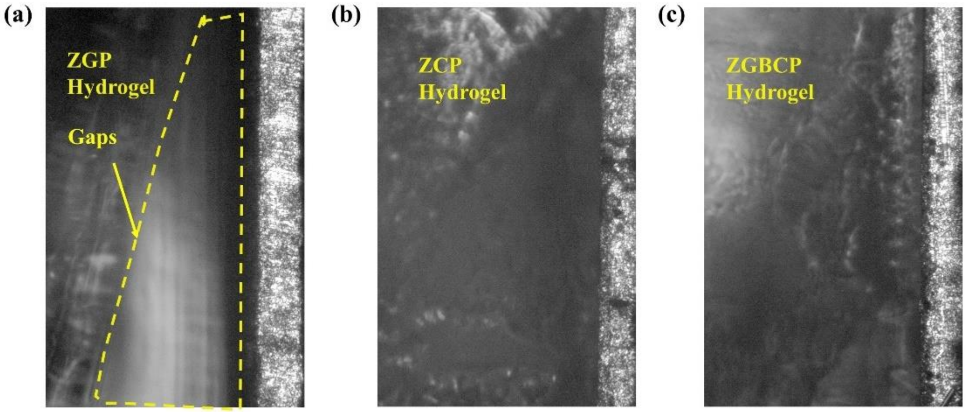

Supplementary Fig. 6 | Optical microscopic images of the interface between different hydrogels **a,** ZGP hydrogel, **b,** ZCP hydrogel, and **c,** ZGBCP hydrogel adhesion to Zn electrode.

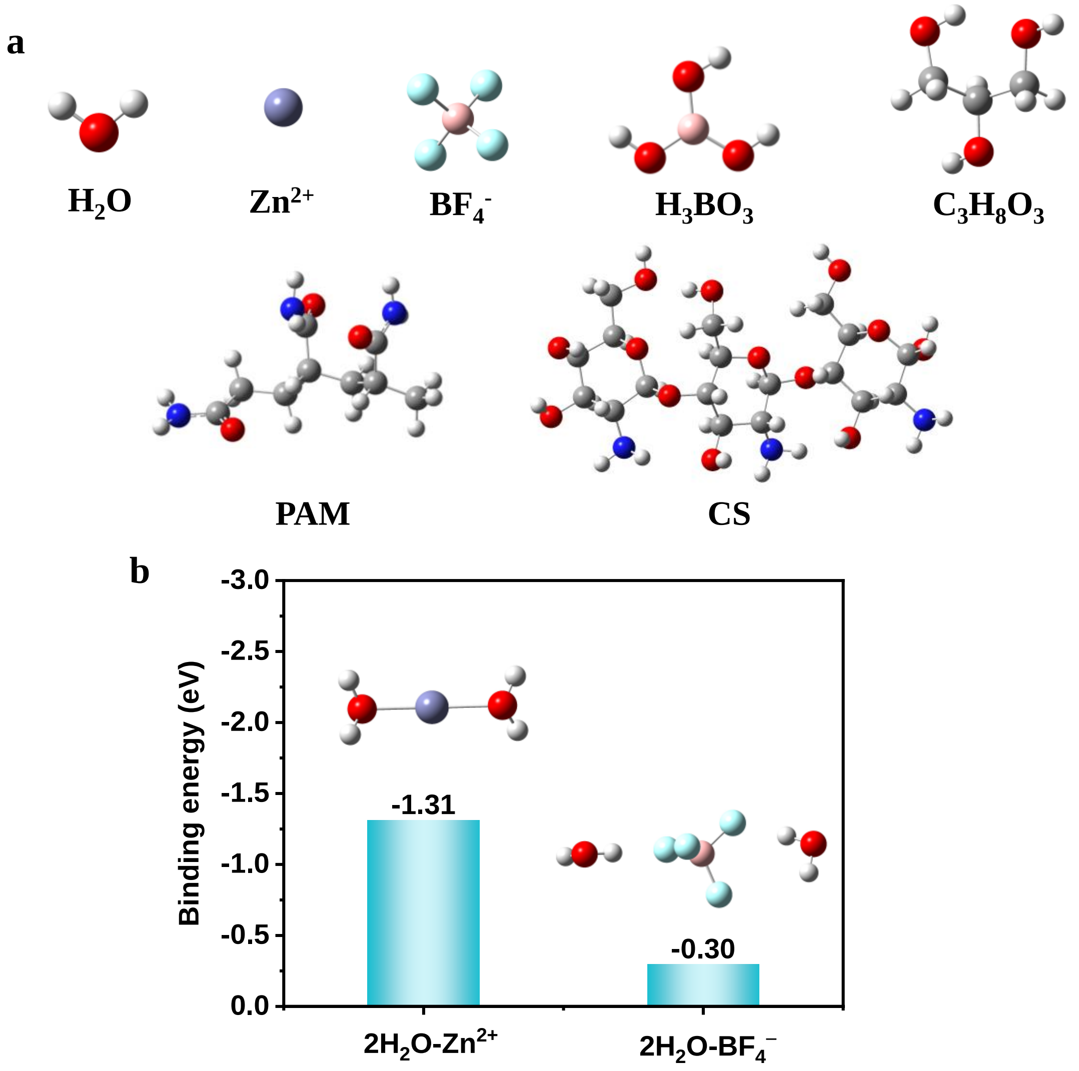

Supplementary Fig. 7 | **a,** Molecule models used in the MD simulations. **b,** Binding energies of Zn^{2+} and BF_4^- with two H_2O molecules.

To avoid comparing absolute energies of mono-dentate (H_2O) and other bidentate complexes involved directly, the binding energies of Zn^{2+} and BF_4^- with two H_2O molecules were provided as Supplementary Fig. 7b. It can be seen that the boric acid, glycerol, PAM, and CS tend to replace the H_2O of the cationic or anionic solvation structures even compared with the binding energies from two H_2O molecules.

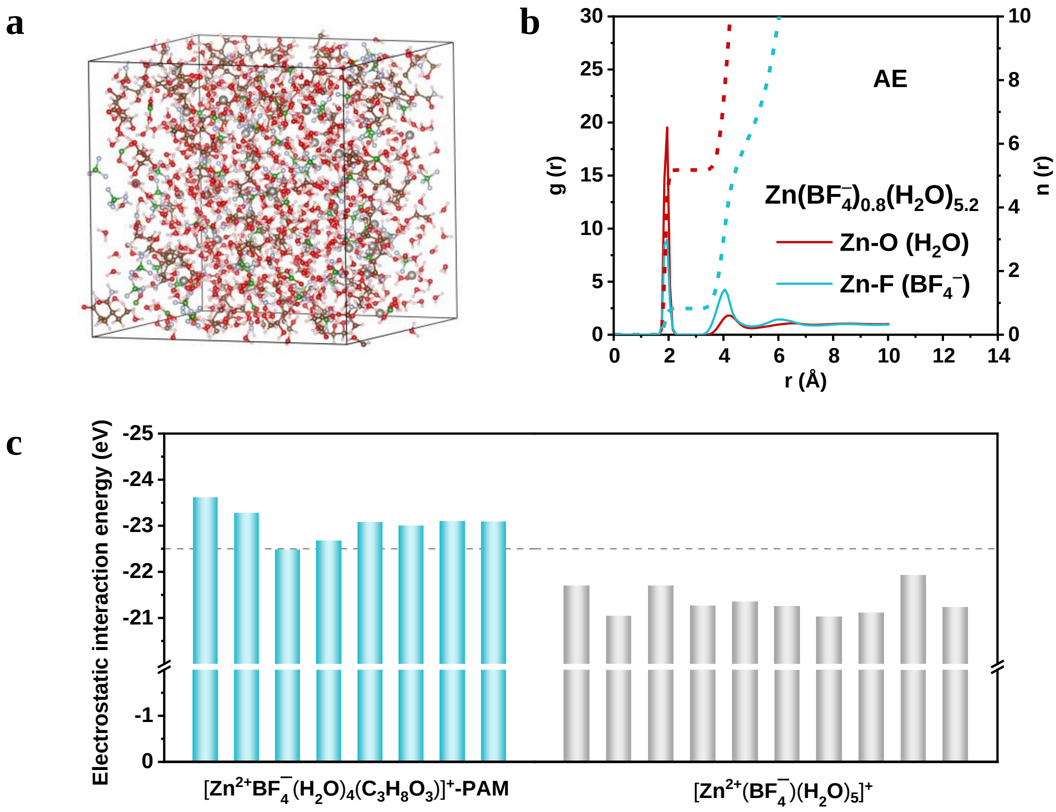

Supplementary Fig. 8 | MD simulation of AE and ZGBCP electrolytes. **a,** MD snapshot of ZGBCP electrolyte. **b,** g(r) and n(r) of the Zn^{2+} in the AE electrolyte. **c,** Electrostatic interaction energy of the H sites in H_2O of $[Zn^{2+}BF_4^{-}(H_2O)_5]^+$ and $[Zn^{2+}BF_4^{-}(H_2O)_4(C_3H_8O_3)\ ]^+$-PAM based on the B97-3c level DFT calculations.

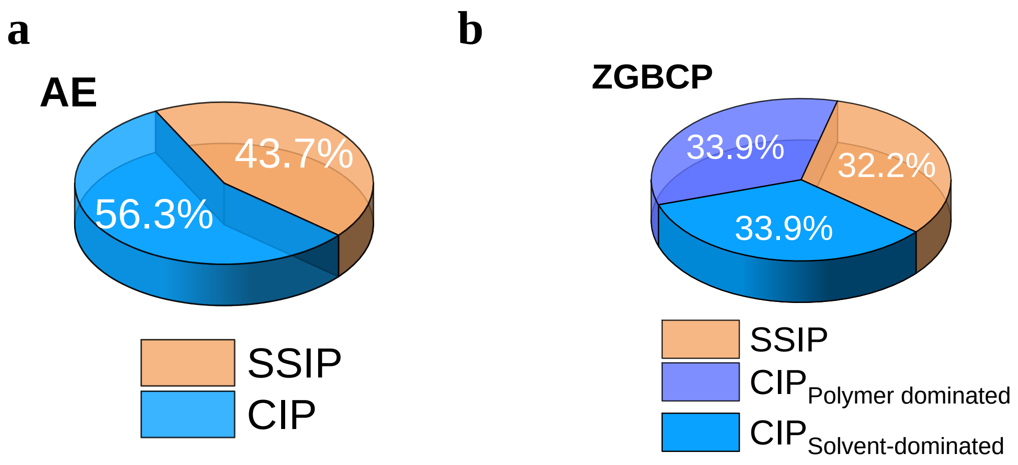

Supplementary Fig. 9 | Distribution of the solvent separated ions pairs (SSIP) and contact ion pairs (CIP) in the **a,** AE, and **b,** ZGBCP electrolyte.

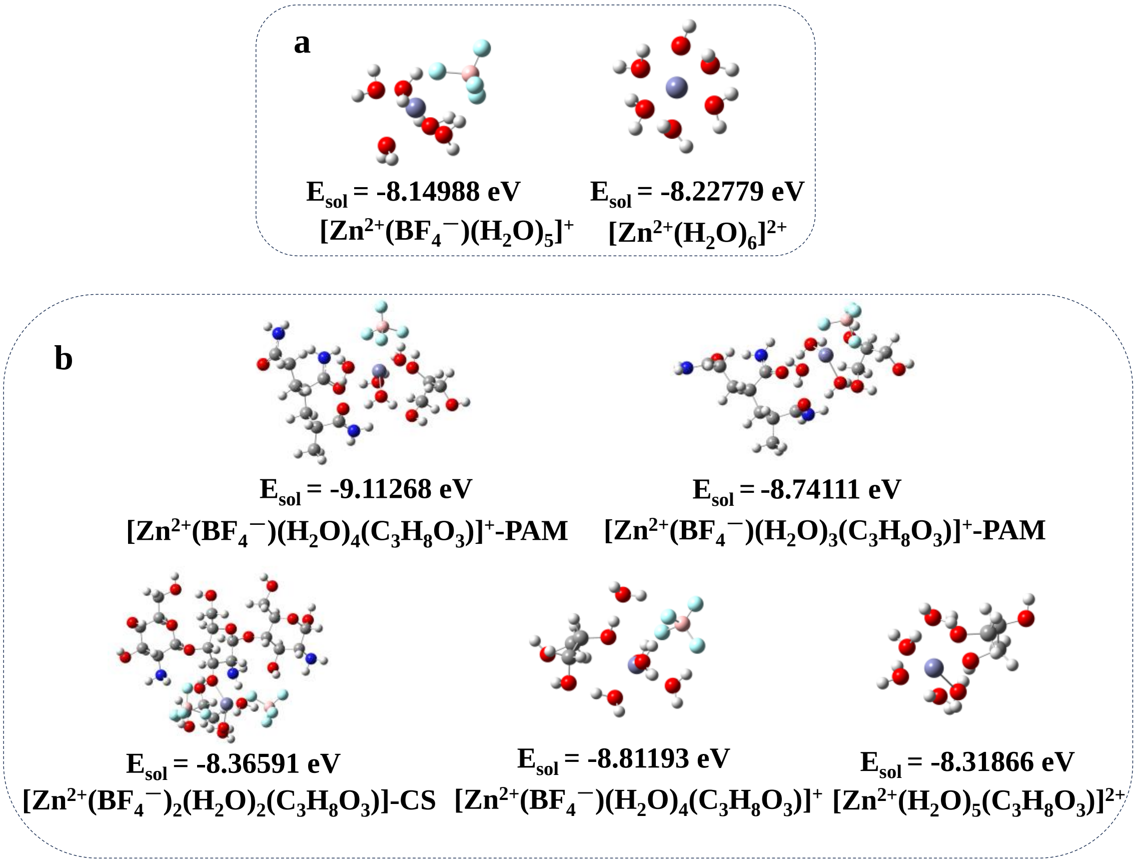

Supplementary Fig. 10 | Various cationic solvation structures obtained from MD simulations and corresponding solvation energy based on the B97-3c level DFT calculations. **a,** $[Zn^{2+}(BF_4^{-})(H_2O)_5]^{+}$ and $[Zn^{2+}(H_2O)_6]^{2+}$ in the AE electrolyte, and **b,** $[Zn^{2+}(BF_4^{-})(H_2O)_4(C_3H_8O_3)]^{+}$-PAM, $[Zn^{2+}(BF_4^{-})(H_2O)_3(C_3H_8O_3)]^{+}$-PAM, $[Zn^{2+}(BF_4^{-})_2(H_2O)_2(C_3H_8O_3)]$-CS, $[Zn^{2+}(BF_4^{-})(H_2O)_4(C_3H_8O_3)]^{+}$, and $[Zn^{2+}(H_2O)_5(C_3H_8O_3)]^{2+}$ in the ZGBCP electrolyte.

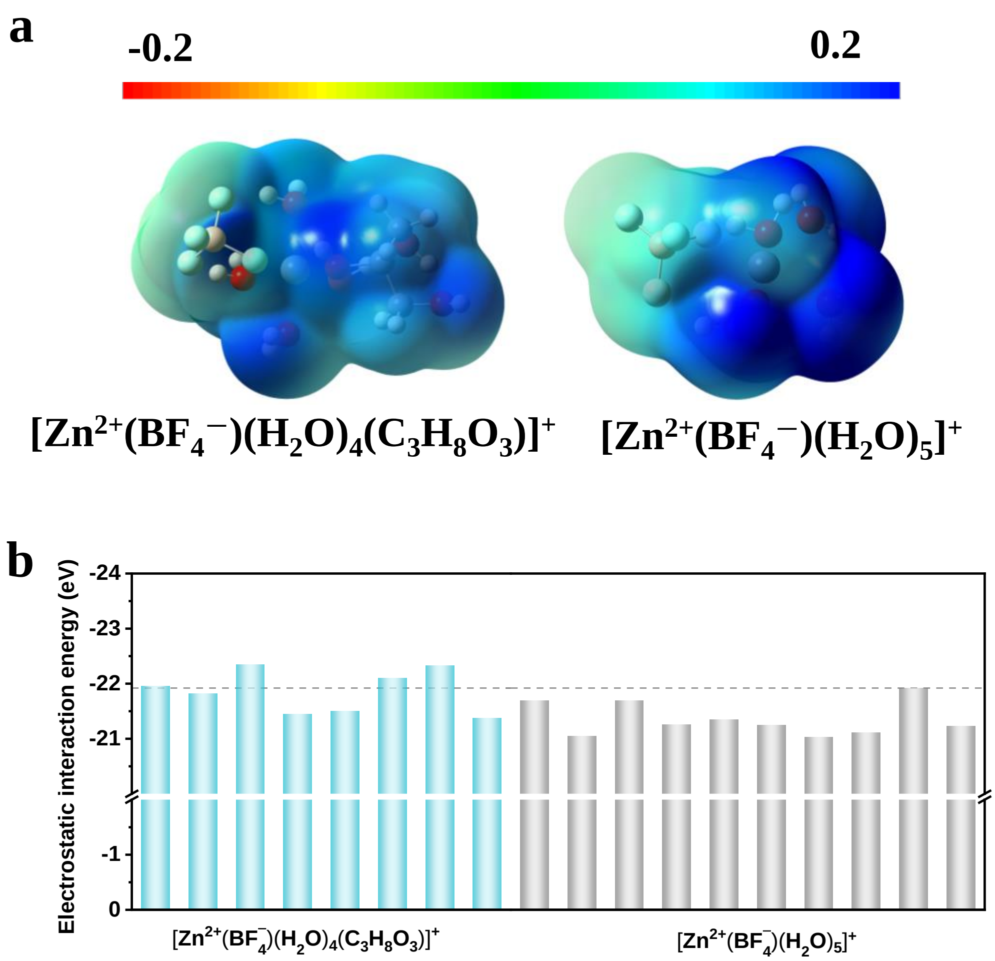

Supplementary Fig. 11 | The CIP-type solvation structure without polymer involved based on the B97-3c level DFT calculations. **a,** The ESP distribution, **b,** Electrostatic interaction energy of the H sites in H_2O of CIP-type cationic solvation structure $[Zn^{2+}BF_4^{-}(H_2O)_4(C_3H_8O_3)]^{+}$ and $[Zn^{2+}(BF_4^{-})(H_2O)_5]^{+}$.

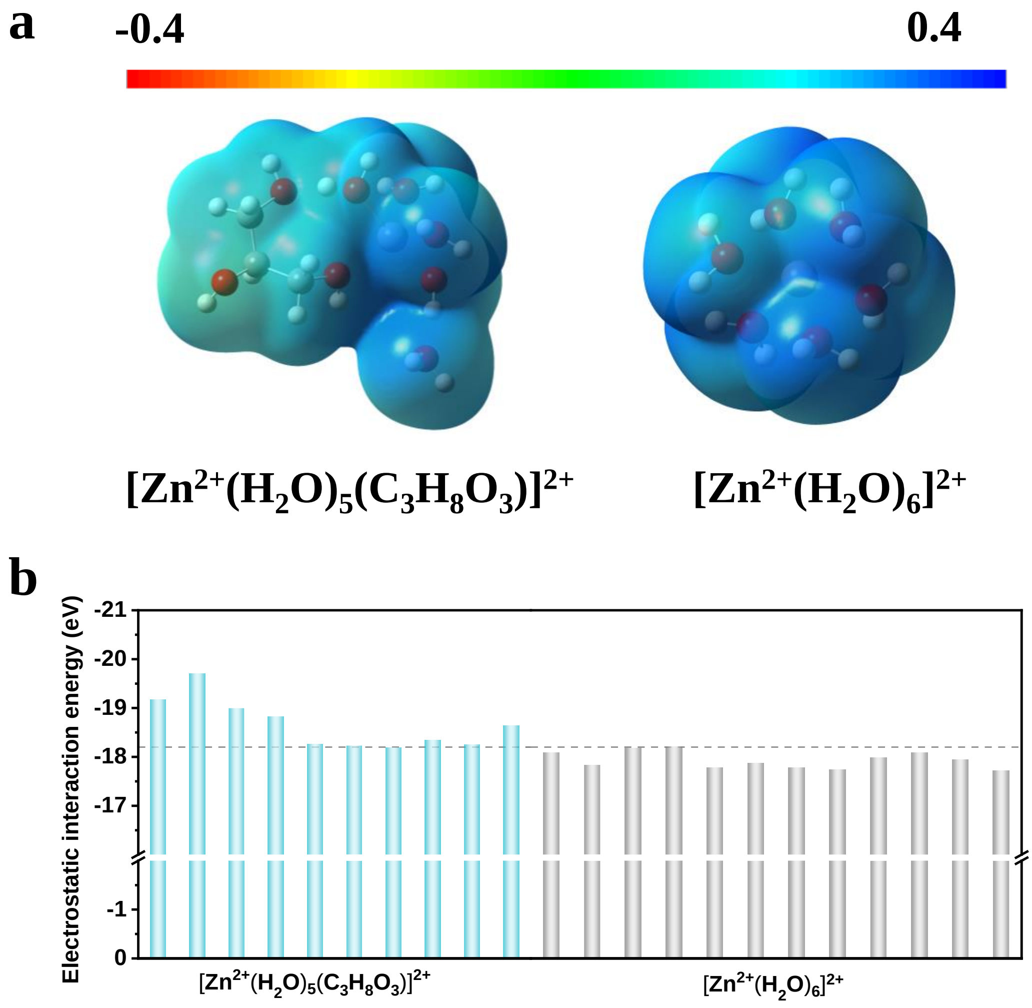

Supplementary Fig. 12 | The SSIP-type solvation structure without polymer involved based on the B97-3c level DFT calculations. **a,** The ESP distribution, **b,** Electrostatic interaction energy of the H sites in H_2O of SSP-type cationic solvation structures $[Zn^{2+}(H_2O)_5(C_3H_8O_3)]^{2+}$ and $[Zn^{2+}(H_2O)_6]^{2+}$.

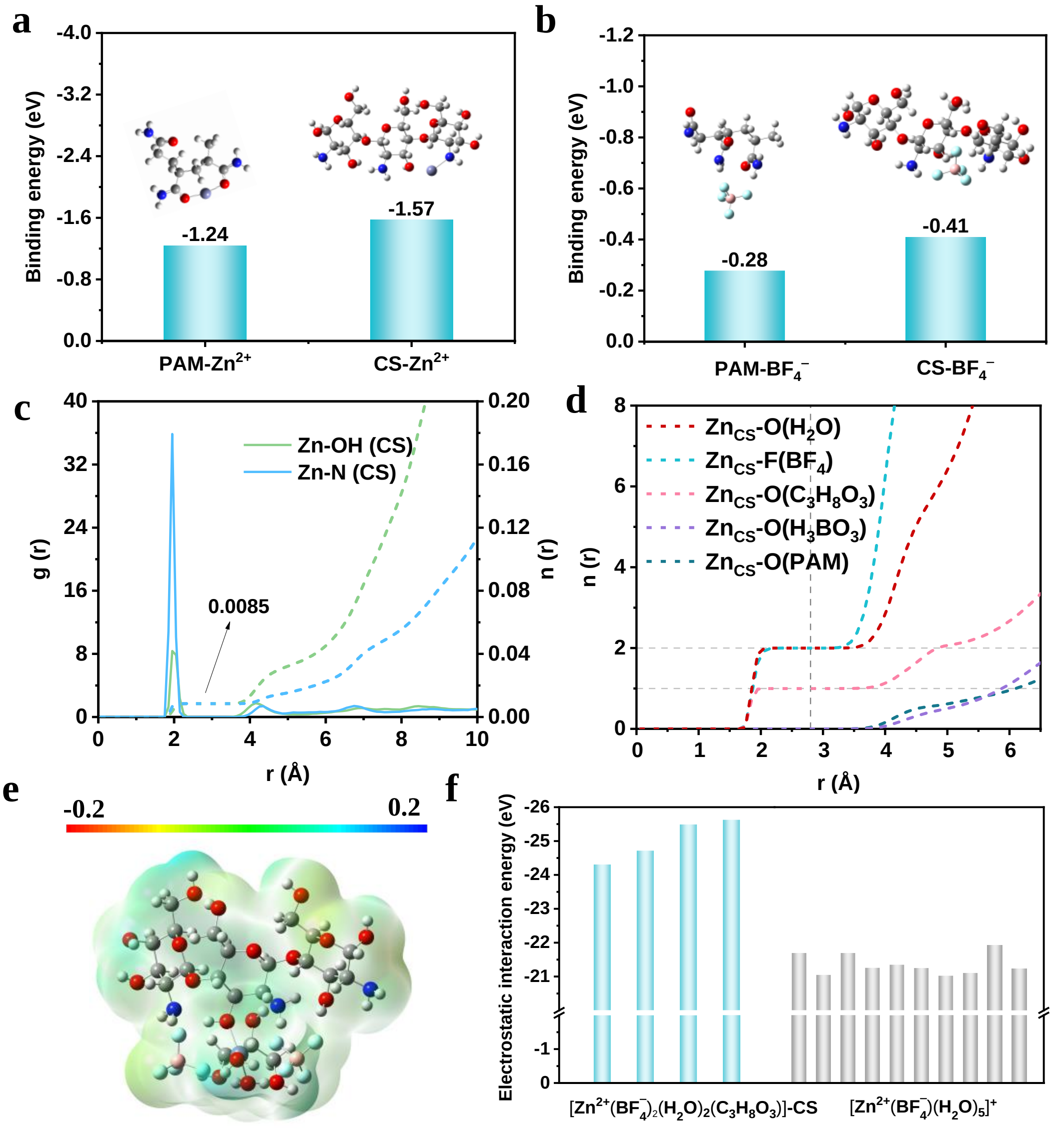

Supplementary Fig. 13 | The cationic solvation structure tuned by the CS **a,** Binding energy of PAM-Zn^{2+} and CS-Zn^{2+} based on the B3LYP/6-311g++(d,p) level DFT calculations. **b,** Binding energy of PAM-BF_4^- and CS-BF_4^- based on the B3LYP/6-311g++(d,p) level DFT calculations. **c,** g(r) and n(r) of overall Zn^{2+} from CS. **d,** n(r) of the Zn^{2+} coordinated by CS nearby. **e,** The ESP distribution, and **f,** Electrostatic interaction energy of the H sites in H_2O of cationic solvation structures $[Zn^{2+}(BF_4^-)_2(H_2O)_2(C_3H_8O_3)]$-CS based on the B97-3c level DFT calculations.

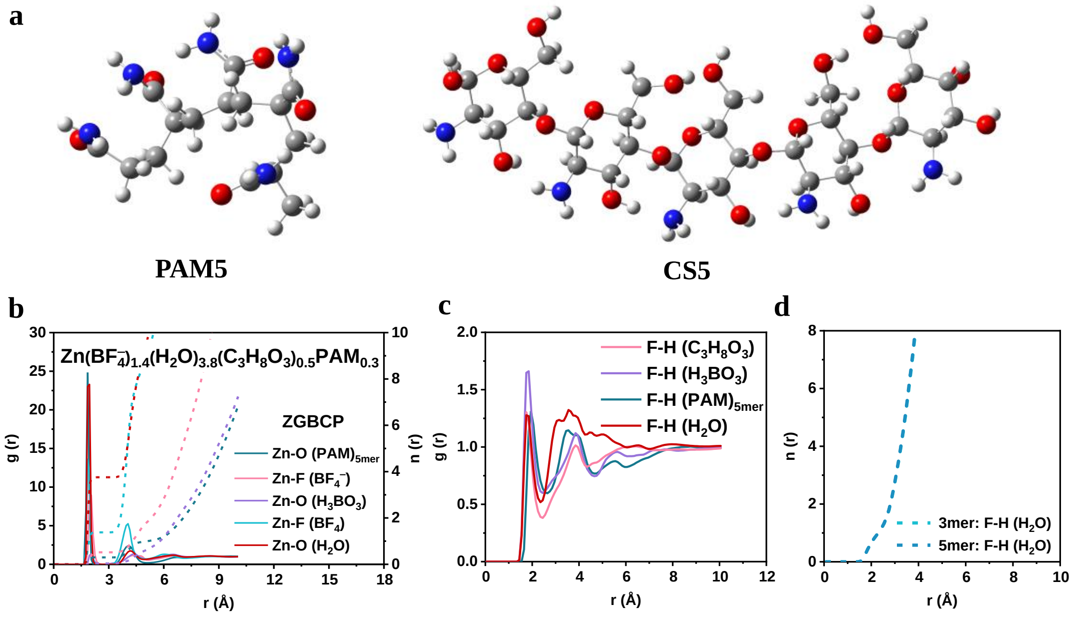

Supplementary Fig. 14 | The MD simulations based on the pentamers models. **a,** Molecular models of PAM and CS pentamers used in the MD simulations. **b,** g(r) and n(r) of Zn^{2+}. **c,** g(r) of F in the ZGBCP_5mer system. **d,** n(r) of F-H (H_2O) in the ZGBCP_3mer and ZGBCP_5mer systems.

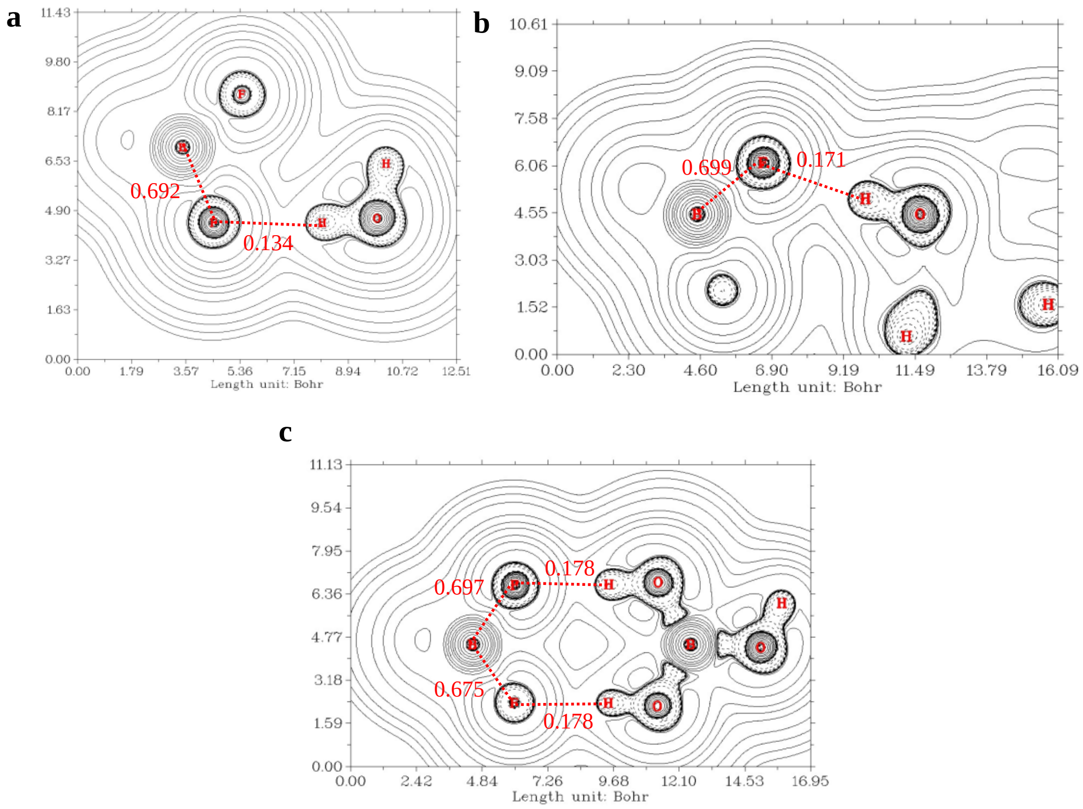

Supplementary Fig. 15 | Laplacian distribution diagrams and mayer bond order for BF_4^- interactions with **a,** H_2O, **b,** $C_3H_8O_3$, and **c,** H_3BO_3 based on the B3LYP/6-311++g(d,p) level DFT calculations.

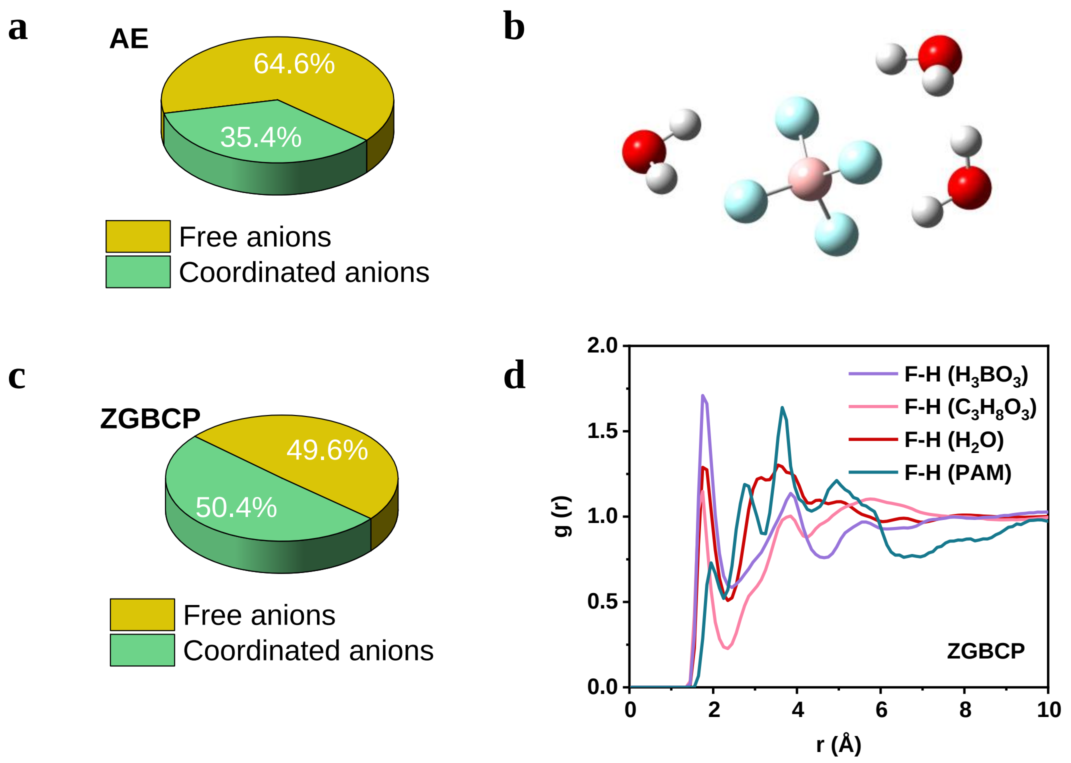

Supplementary Fig. 16 | Distribution and structures of free and coordinated BF_4^-. **a,** Distribution of the free and coordinated BF_4^- in the AE. **b,** The solvation structure of free BF_4^- in the AE. **c,** Distribution of the free and coordinated BF_4^- in the ZGBCP electrolyte. **d,** The g(r) of BF_4^- in the ZGBCP electrolyte. **e,**

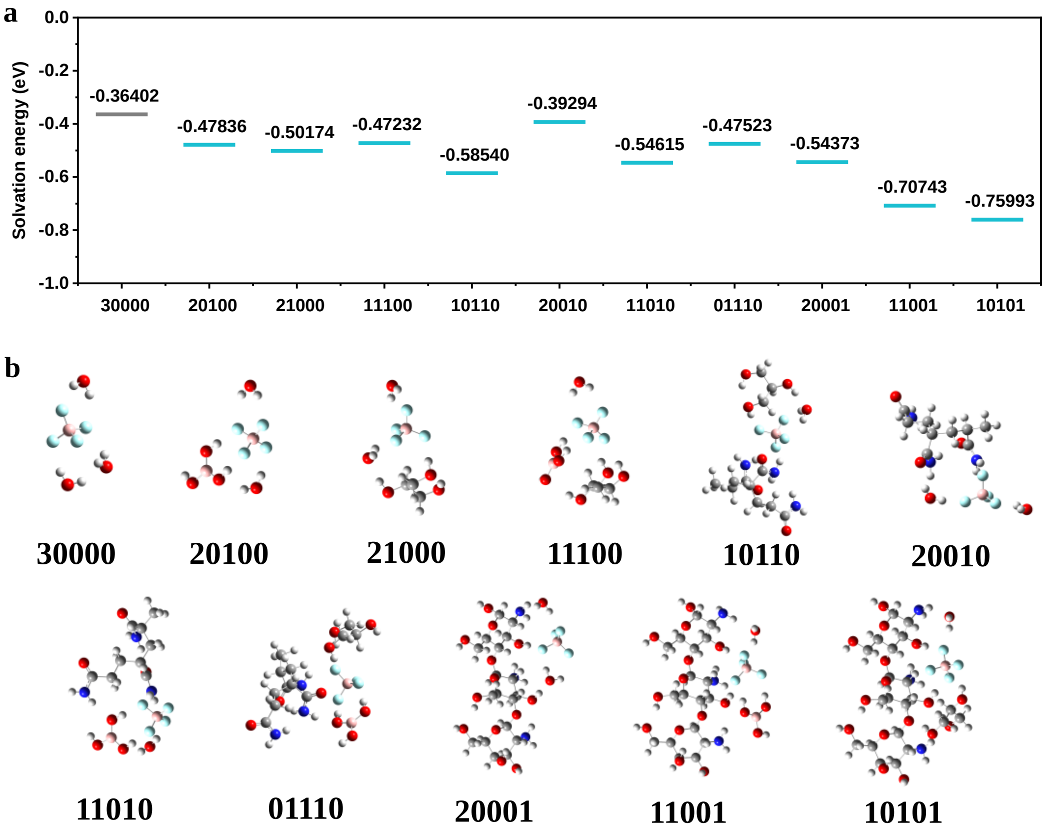

Supplementary Fig. 17 | a, Energy level, and **b,** optimized models of different potential solvation structures of free anions denoted as $BF_4^-(H_2O)_a(C_3H_8O_3)_b(H_3BO_3)_c(PAM)_d(CS)_e$ based on the B97-3c level DFT calculations.

For the solvation behavior of free anions tuned by the CRACSS strategies, the 10101 exhibited the highest solvation energy due to the more abundant hydrogen-bond donors (-NH_2, -OH) of CS than PAM with only -$CONH_2$ donors. While in the presence of H_2O and PAM, the single coordination sites from PAM make their interaction with BF_4^- relatively weak. Meanwhile, due to the presence of PAM, the coordination of H_2O will be limited, resulting in a less stable solvation structure than the strong coordination from glycerol and CS. Notably, all of these structures are important for the interfacial stabilization of Zn anodes and desolvation process for the PANI cathodes.

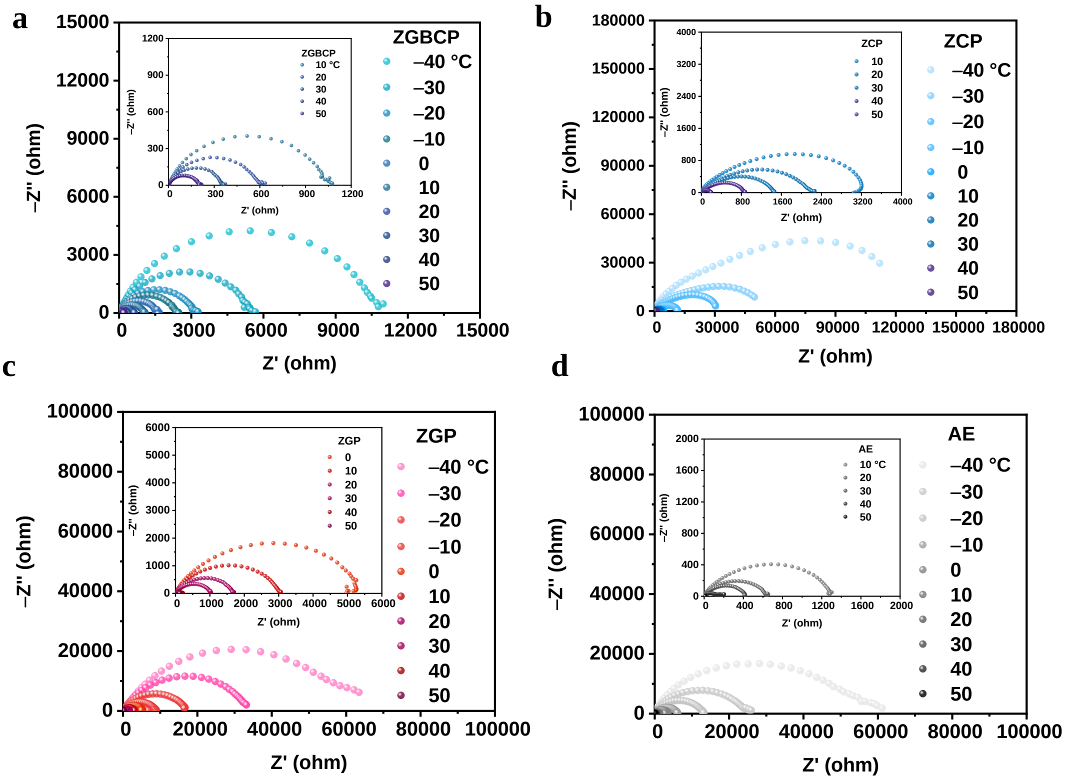

Supplementary Fig. 18 | The temperature-dependent EIS tests of the Zn||Zn symmetric cells using **a,** ZGBCP, **b,** ZCP, **c,** ZGP, and **d,** AE electrolytes.

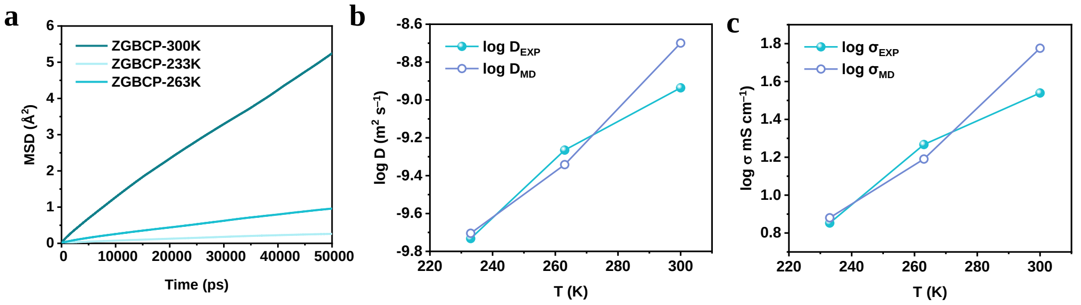

Supplementary Fig. 19 | Ionic transport property calculated by the MD simulation under different temperatures. **a,** MSD of Zn^{2+} obtained from MD simulations under different temperatures. **b,** diffusion coefficients and **c,** conductivities of ZGBCP electrolytes based on simulation and experiments under different temperatures.

To investigate the diffusivity of the ZGBCP electrolyte, MD simulations of the ZGBCP system at different temperatures were further conducted. Based on the MSD calculations (Supplementary Fig. 18), the theoretical diffusion coefficients σ at different temperatures can be obtained. As seen from Supplementary Fig. 18b, the diffusion coefficients and conductivities obtained from the simulations exhibit similar trends and orders of magnitudes as those obtained from the experimentally measured conductivity versus temperatures as shown in Supplementary Fig.18c This confirms the accuracy and reliability of the simulation results. The diffusion coefficients were calculated based on the formula (1):

$$D = MSD/6t \qquad (1)$$

, where the MSD is the Mean Square Displacement obtained from MD simulations, the t is the simulation time.

The simulated conductivities were calculated based on the Nernst-Einstein equation (2):

$$\sigma = D \cdot \frac{nq^2}{K_B T} \qquad (2)$$

, where the n is the concentration of Zn^{2+}, the q is the charge amount of Zn^{2+}, K_B is the Boltzmann's constant, and the T is the temperature.

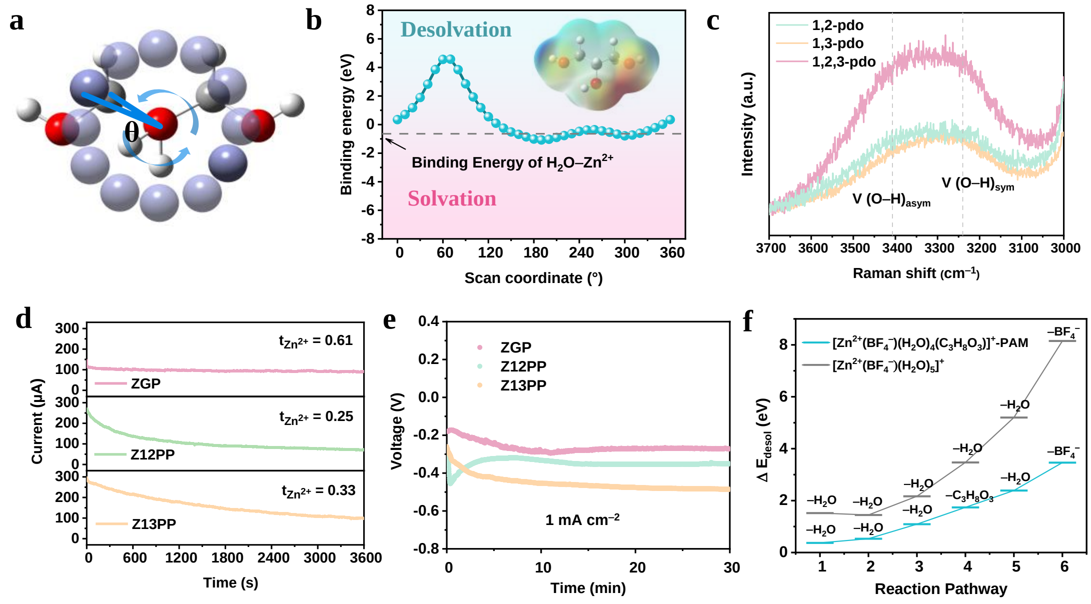

Supplementary Fig. 20 | The analysis of acceleration effect of desolvation tuned by the glycerol. **a,** Schematic diagram of rigid scan of Zn^{2+} rotating around glycerol molecule at a Zn-O-C-C dihedral angle θ. **b,** Binding energy between the Zn^{2+} and glycerol based on the rigid scan with B3LYP/6-311++g(d,p) level DFT calculation. **c,** Raman spectra of the pure glycerol, 1,2-dipropanol, and 1,3-dipropanol. **d,** Chronoamperometry tests. **e,** Voltage-time profiles of Zn||Zn symmetrical batteries using ZGP, Z12PP, and Z13PP electrolytes. **f,** Desolvation energy barrier $\triangle E_{desol}$ of $[Zn^{2+}(BF_4^-)(H_2O)_4(C_3H_8O_3)]^+$-PAM and $[Zn^{2+}(BF_4^-)(H_2O)_5]^+$ obtained from the DFT calculation based on the B97-3c level DFT calculations.

To further support the hypothesis, the roles of similar compounds such as 1,2-propanediol and 1,3-propanediol with different intramolecular hydrogen bonding in regulating the desolvation were systematically investigated. As seen from Supplementary Fig. 19c, Raman spectroscopy reveals that the intensity of the symmetric and asymmetric stretching vibrations of O−H bonds in glycerol is much higher than those of 1,2-propanediol and 1,3-propanediol, indicating the stronger coupling of O−H vibrational modes caused by more intense intramolecular hydrogen bonding of glycerol[1]. Furthermore, to identify the influence of the intramolecular

hydrogen bonding on the desolvation process, CA test of the Zn||Zn cells with the electrolytes containing glycerol, 1,2-propanediol and 1,3-propanediol were conducted. It was observed from Supplementary Fig. 19d that the Zn^{2+} transfer number of ZGP electrolyte with glycerol is 0.61, whereas for electrolytes (Z12PP and Z13PP) using 1,2-propanediol and 1,3-propanediol, the Zn^{2+} transfer number are only 0.25 and 0.33, respectively, proving that glycerol did accelerate the Zn desolvation as well as Zn plating reaction. Due to the accelerated desolvation process, Zn||Zn symmetric cell using ZGP electrolyte exhibits the lowest initial plating polarization voltage at a current density of 1 mA cm^{-2}, shown in Supplementary Fig. 19e. What's more, as illustrated in the Supplementary Fig. 19f, the desolvation energy barrier of the solvation structure $[Zn^{2+}(BF_4^-)(H_2O)_4(C_3H_8O_3)]^+$-PAM is only 3.463 eV based on the DFT calculations, which shows a dramatic decrease compared with the $[Zn^{2+}(BF_4^-)(H_2O)_5]^+$ (8.150 eV).

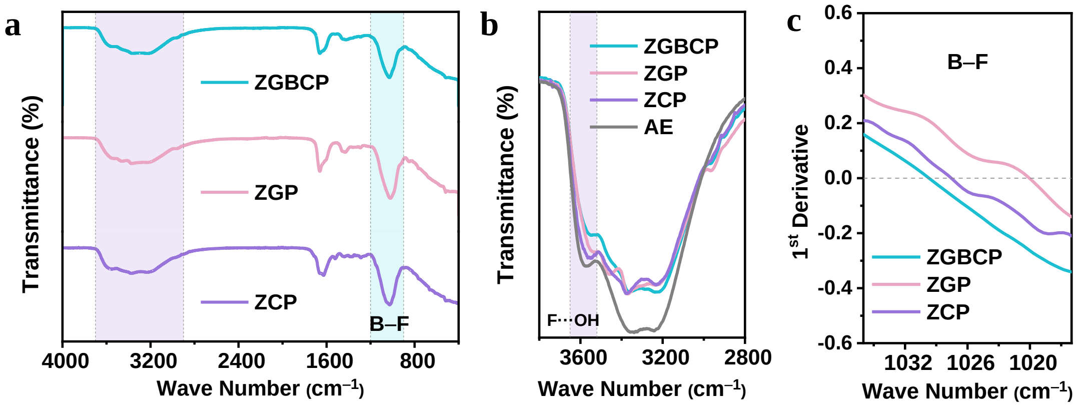

Supplementary Fig. 21 | The spectral characterizations of the different electrolytes. **a,** ATR-FT-IR spectra, and corresponding **b,** Local enlarge, and **c,** First order differentiation.

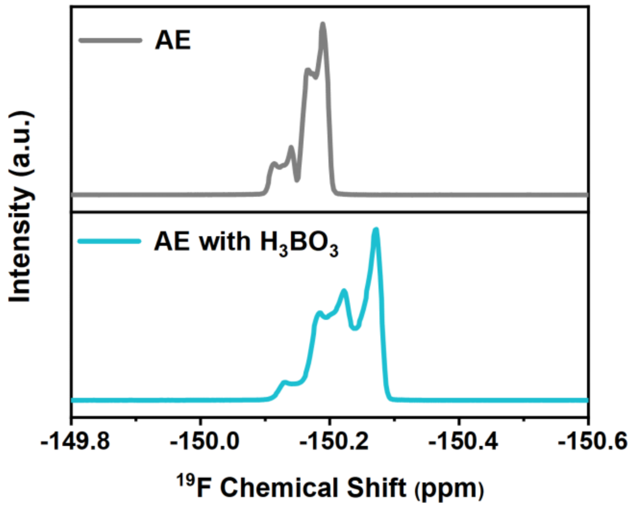

Supplementary Fig. 22 | ^{19}F NMR spectra of AE electrolyte added with/without boric acid.

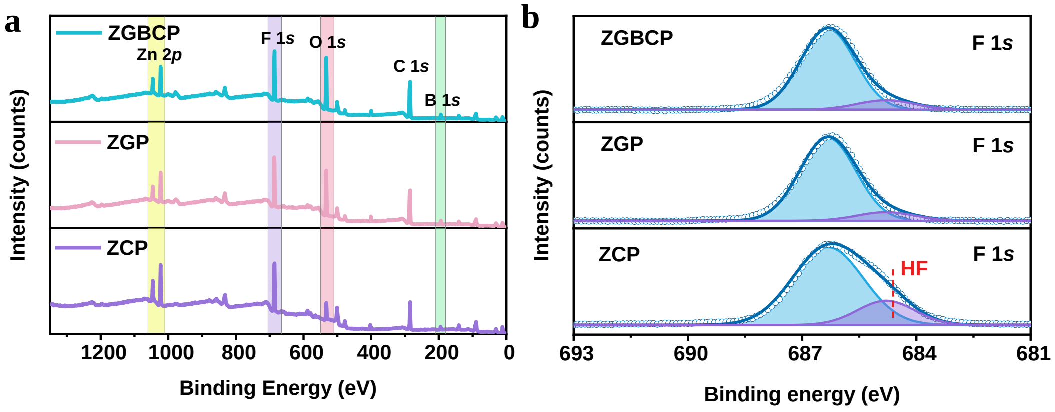

Supplementary Fig. 23 | a, The full spectra and **b,** XPS F 1*s* spectra of ZGBCP, ZGP, and ZCP electrolytes.

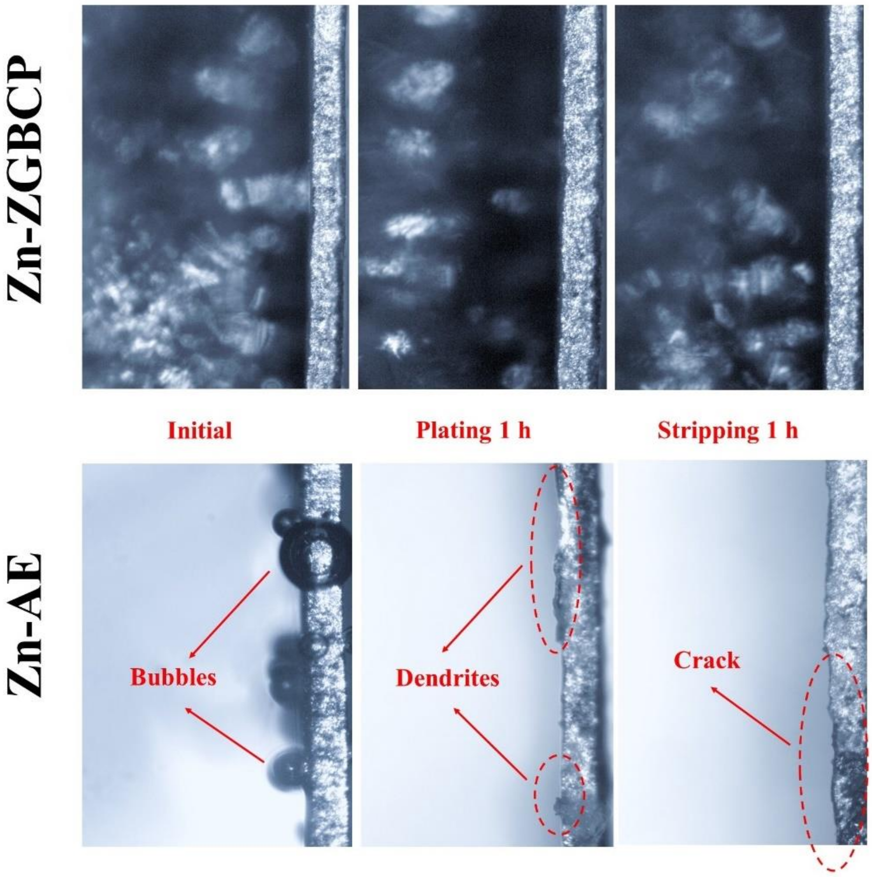

Supplementary Fig. 24 | The in-situ optical observation of the Zn plating and stripping reaction at the interface between Zn and electrolytes (top: ZGBCP electrolyte; bottom: AE electrolyte).

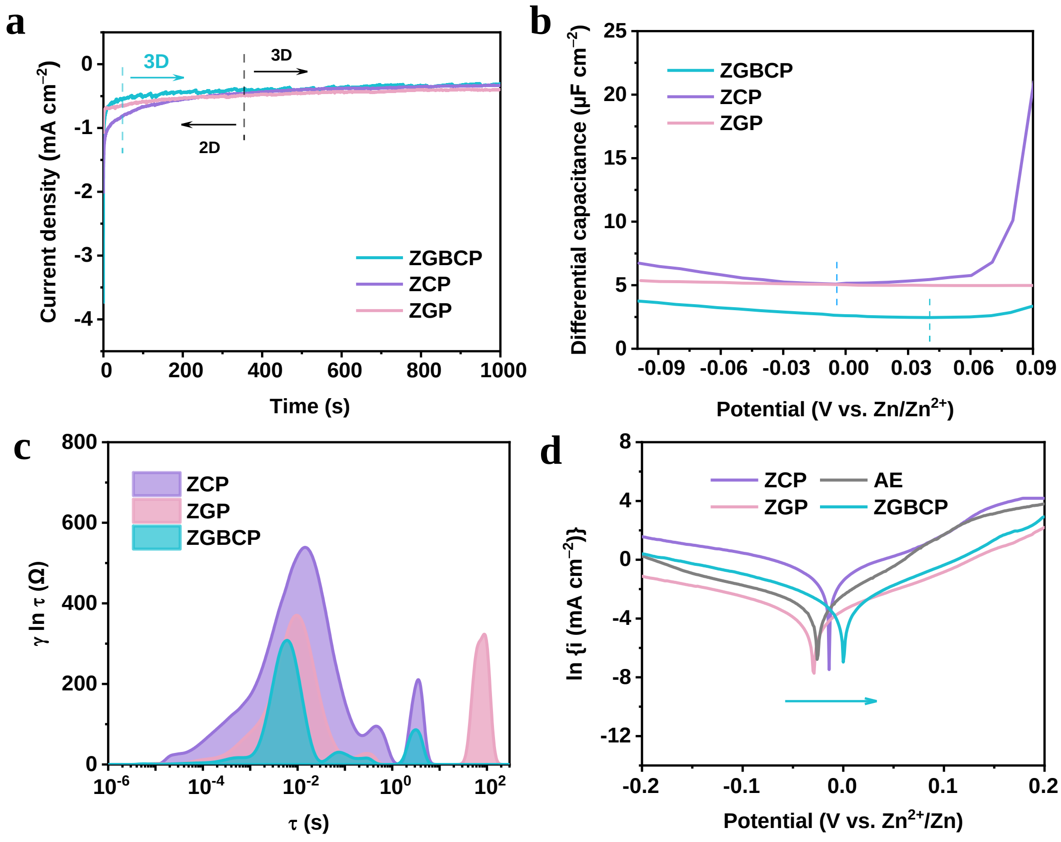

Supplementary Fig. 25 | The electrochemical analysis of the reaction kinetics of the Zn anodes using different electrolytes. **a,** Chronoamperometry curves under polarization potential of −20 mV. **b,** Differential capacitance curves. **c,** DRT analysis of the Zn||Zn symmetric cells. **d,** Tafel tests for the reaction kinetics of Zn anodes using different electrolytes.

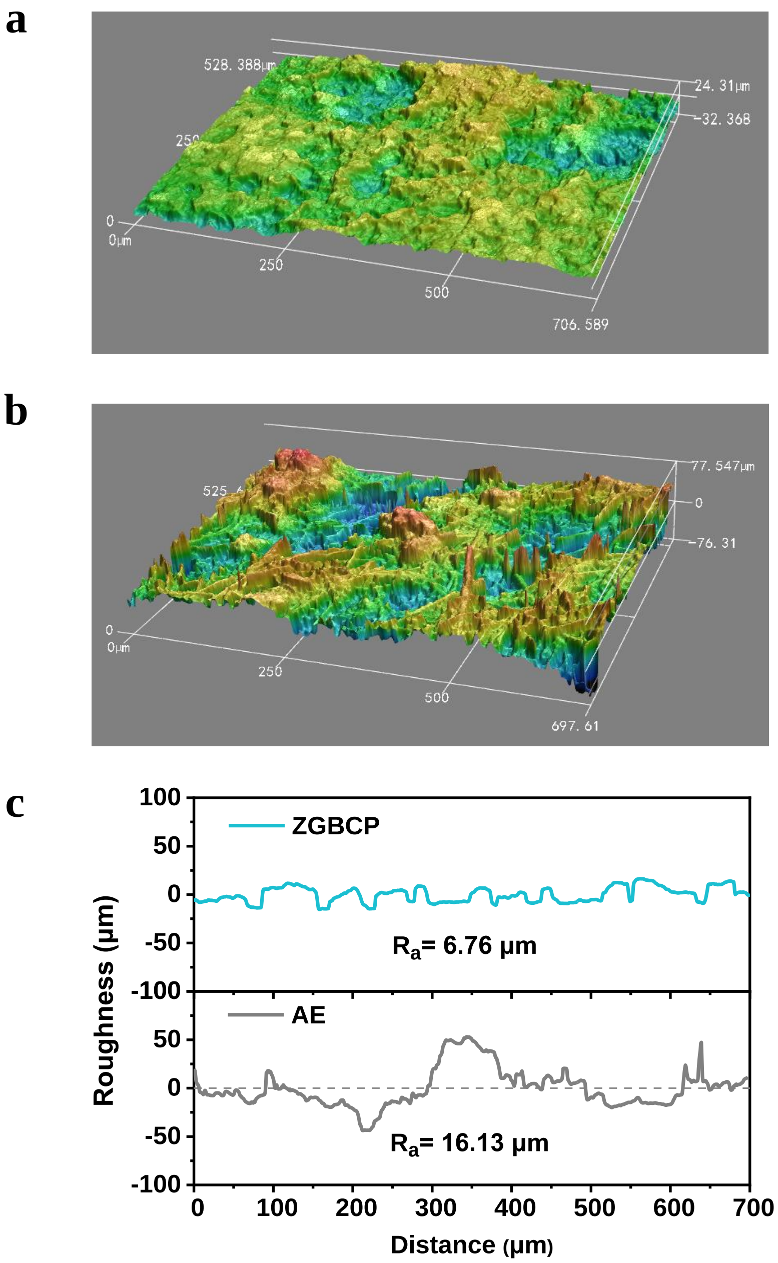

Supplementary Fig. 26 | Optical profile reconstruction of the anodes using **a,** ZGBCP and **b,** AE electrolyte and **c,** roughness tests of the cycled anodes using different electrolytes.

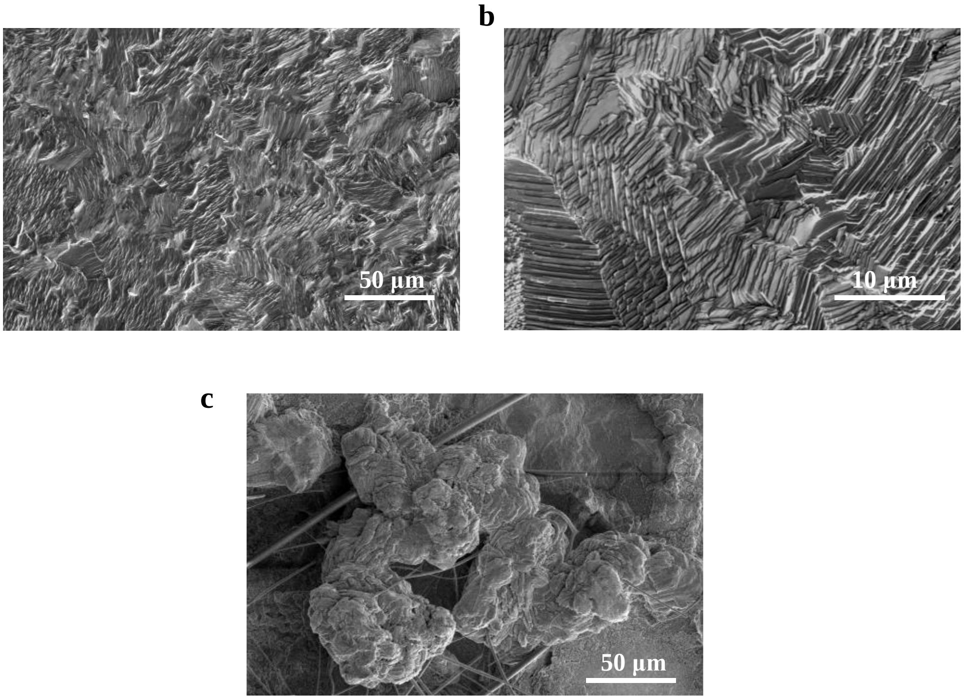

Supplementary Fig. 27 | SEM images of the Zn anodes after 50 cycles using **a-b,** ZGBCP. **c,** AE electrolyte.

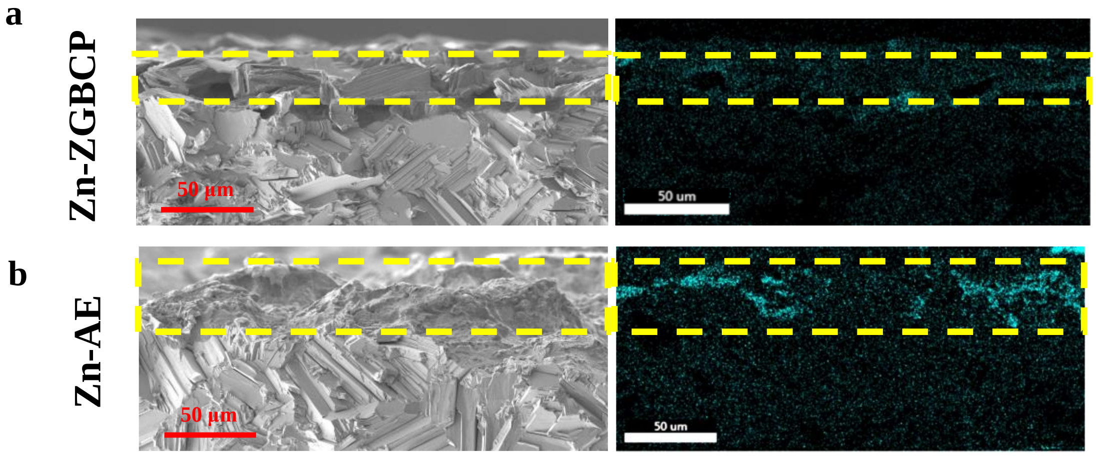

Supplementary Fig. 28 | SEM images corresponding EDS mappings of the cross-sections of Zn anodes using **a,** ZGBCP and **b,** AE electrolytes.

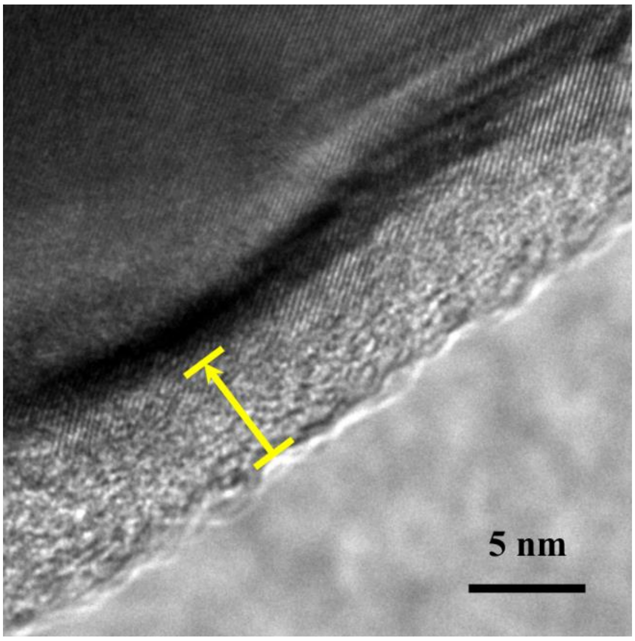

Supplementary Fig. 29 | TEM images of the cross-section of the Zn anodes using ZGBCP electrolytes after 10 cycles.

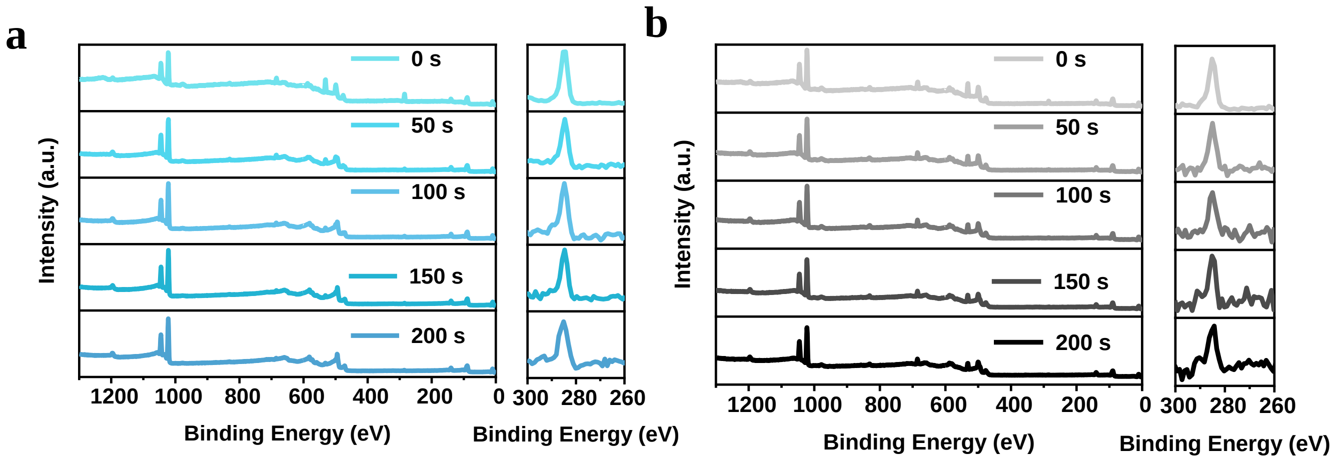

Supplementary Fig. 30 | The XPS survey and corresponding C 1*s* spectra of Zn anodes at different depths using **a,** ZGBCP, and **b,** AE electrolyte.

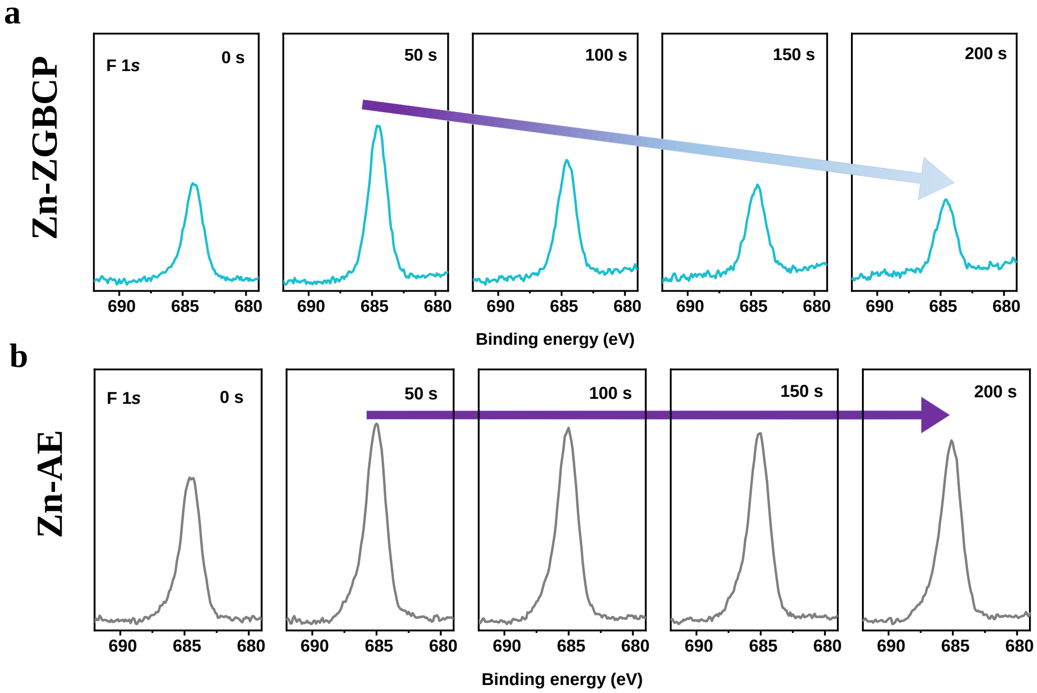

Supplementary Fig. 31 | XPS F 1*s* spectra of the cycled Zn anodes surface in different depth using **a,** ZGBCP, and **b,** AE electrolyte.

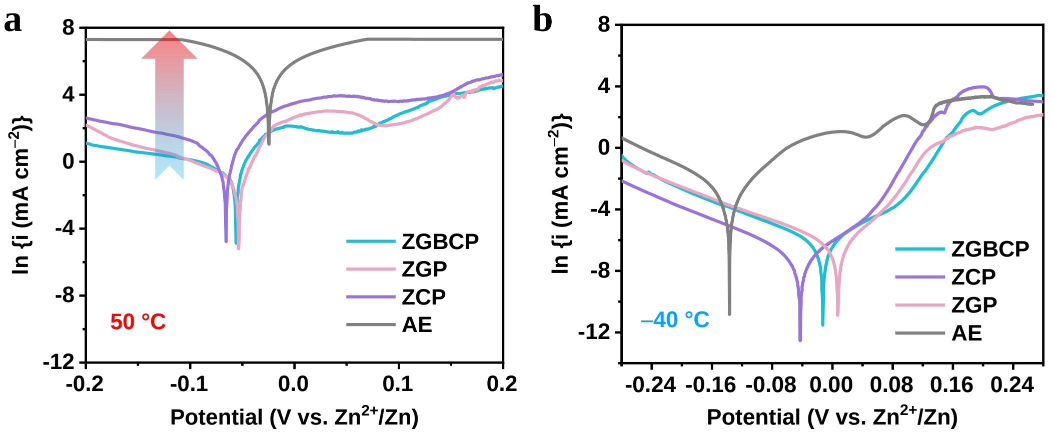

Supplementary Fig. 32 | Stability and kinetics of stripping/plating reaction characterized by Tafel tests using different electrolytes under **a,** -40 °C, and **b,** 50 °C.

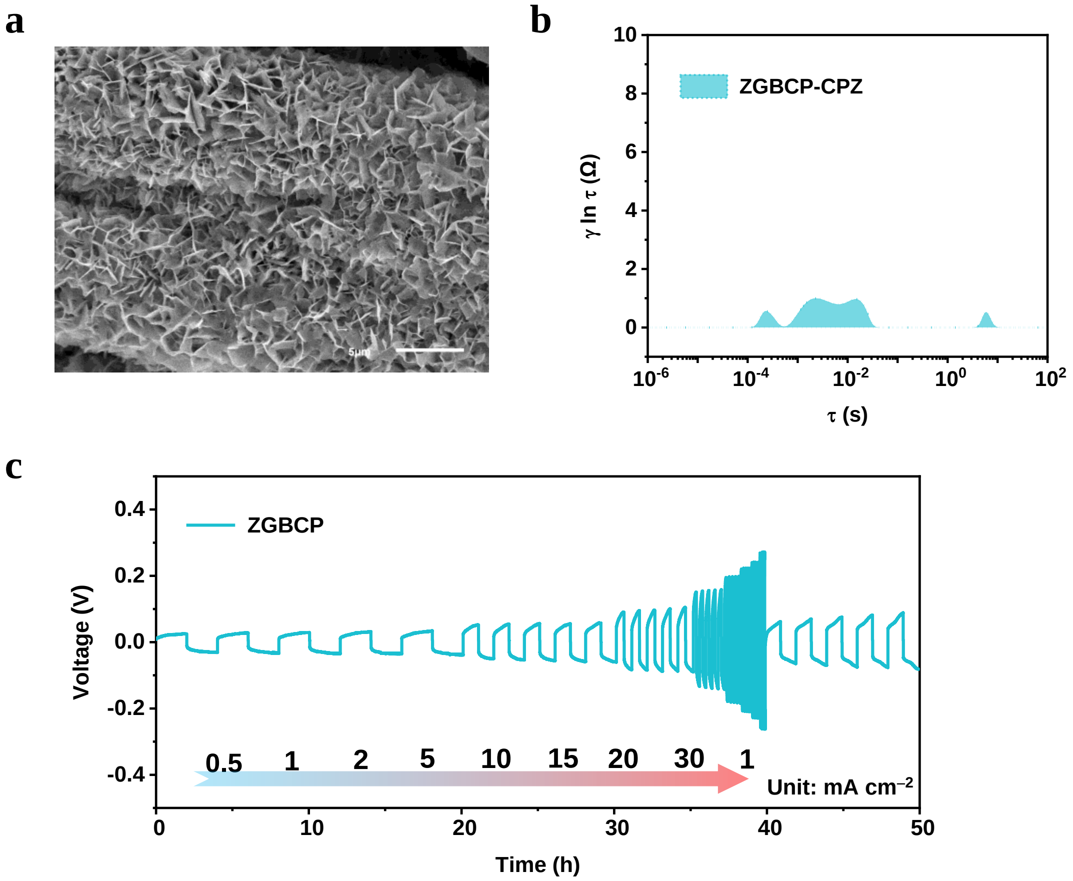

Supplementary Fig. 33 | The characterizations of CPZ electrodes. **a,** SEM image. **b,** DRT analysis and **c,** rate performance of CPZ||CPZ symmetric cell using the ZGBCP electrolyte.

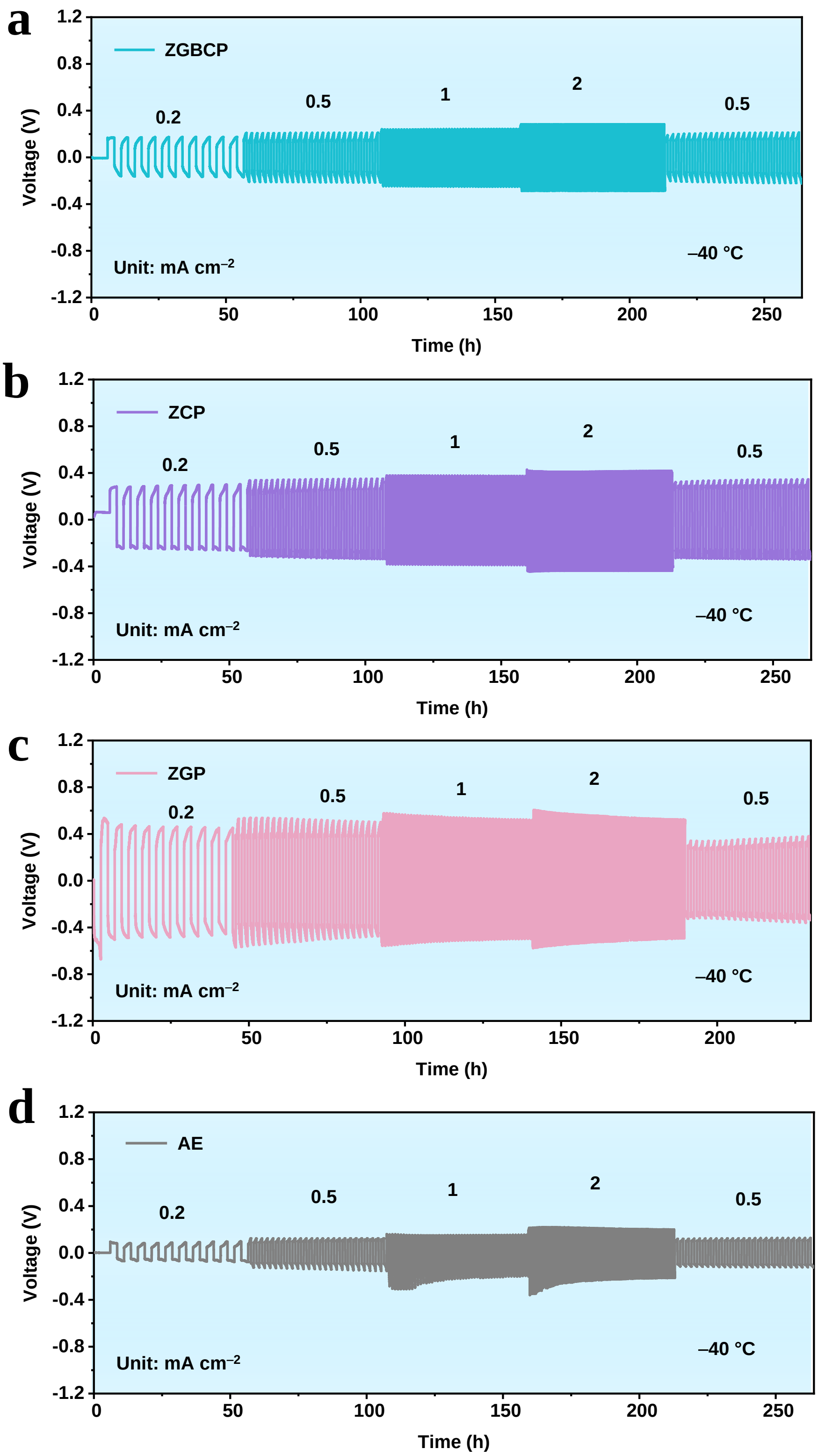

Supplementary Fig. 34 | The rate performance of the Zn||Zn symmetric batteries using **a,** ZGBCP, **b,** ZCP, **c,** ZGP, and **d,** AE electrolytes under -40 °C.

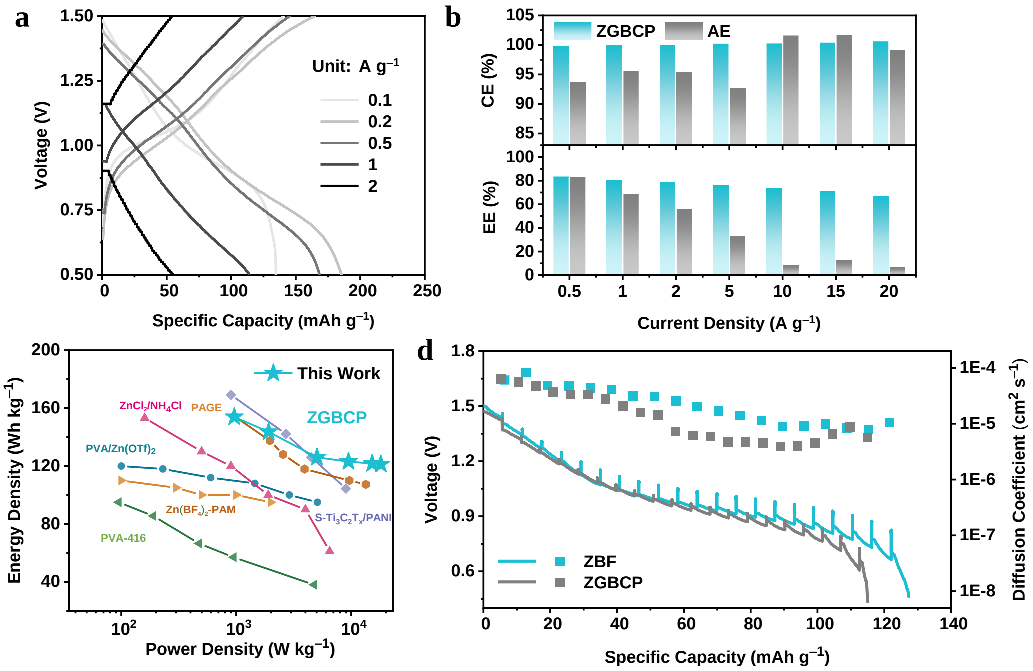

Supplementary Fig. 35 | Kinetics analysis and comparison of the full batteries using PANI cathodes. **a,** GCD profiles of the full batteries using AE electrolyte. **b,** Average CE and average EE under different current densities. **c,** Ragone plots of the full batteries of this work compared with reported works using PANI cathodes[2-7]. **d,** GITT tests using the AE, and ZGBCP electrolytes.

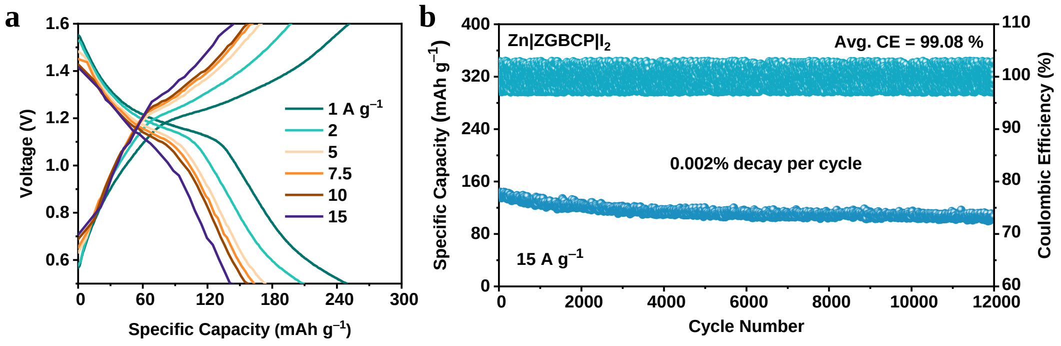

Supplementary Fig. 36 | Rate and cycling performance of the Zn|ZGBCP|I_2 full cell. **a,** GCD profiles under different current densities. **b,** cycling performance at 15 A g^{-1}.

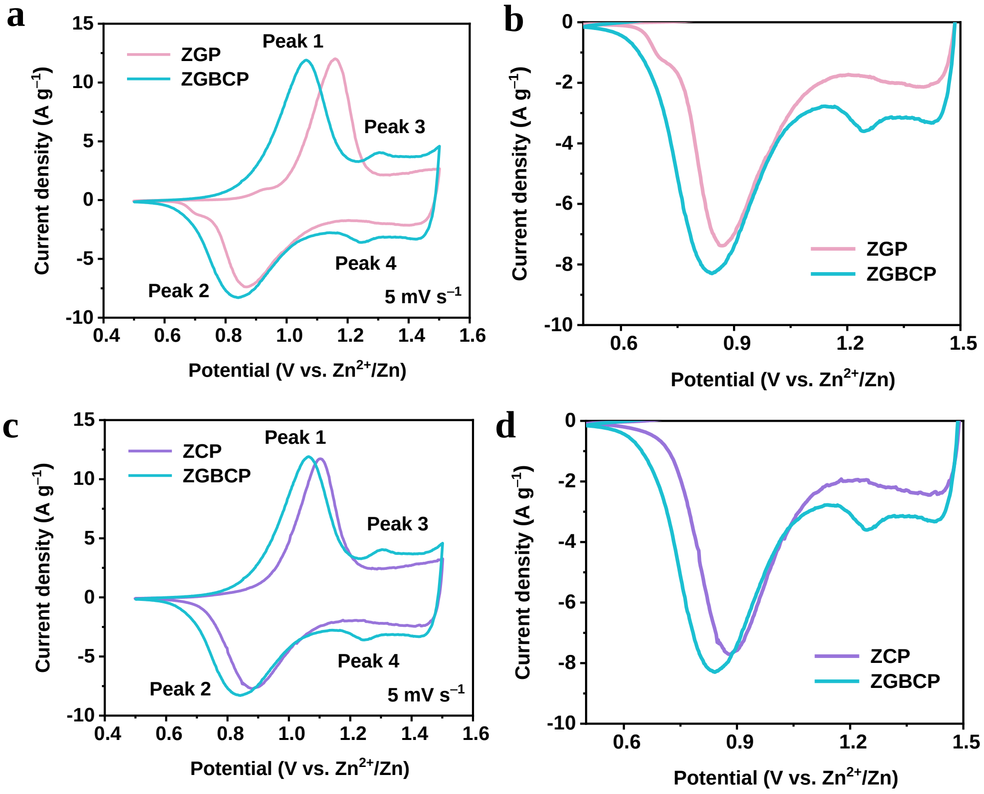

Supplementary Fig. 37 | CV profiles of the Zn||PANI full batteries using **a-b,** ZGP, and **c-d,** ZCP electrolytes.

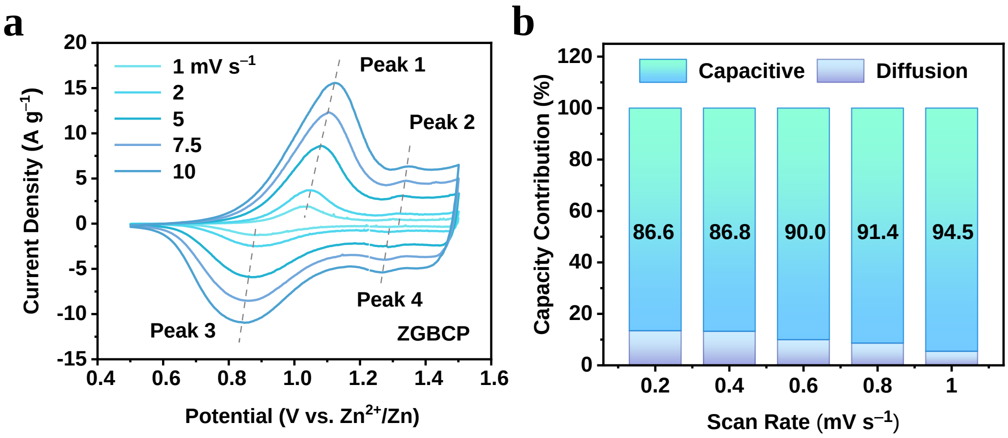

Supplementary Fig. 38 | Reaction kinetics analysis of the Zn|ZGBCP|PANI full cells. **a,** Variable sweep rate (v, 1-10 mV s^{-1}) CV curves. **b,** Proportion of pseudocapacitance contribution using ZGBCP electrolyte at different scanning speeds from 0.2 to 1 mV s^{-1}.

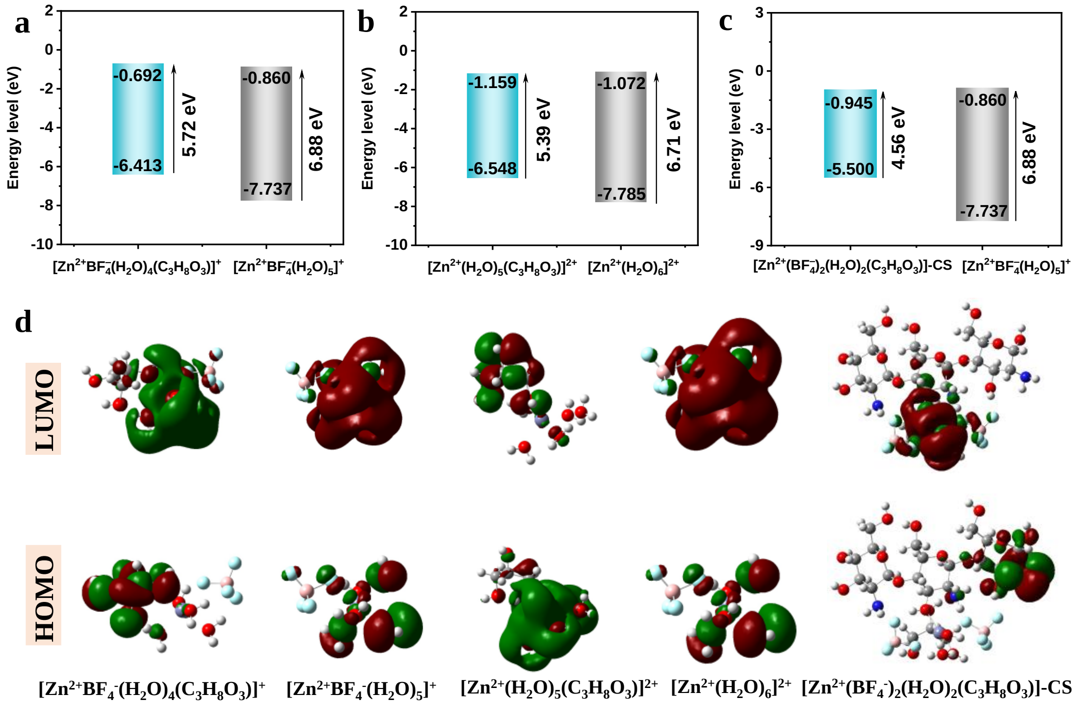

Supplementary Fig. 39 | Energy level of different cationic solvation structures in the AE (right) and ZGBCP (left) electrolytes, respectively based on the B97-3c level DFT calculations. **a,** $[Zn^{2+}(BF_4^-)(H_2O)_4(C_3H_8O_3)]^+$ and $[Zn^{2+}(BF_4^-)(H_2O)_5]^+$, **b,** $[Zn^{2+}(H_2O)_5(C_3H_8O_3)]^{2+}$ and $[Zn^{2+}(H_2O)_6]^{2+}$, **c,** $[Zn^{2+}(BF_4^-)_2(H_2O)_2(C_3H_8O_3)]$-CS and $[Zn^{2+}(BF_4^-)(H_2O)_5]^+$. **d**, The LUMO and HOMO orbitals of the cationic solvation structures.

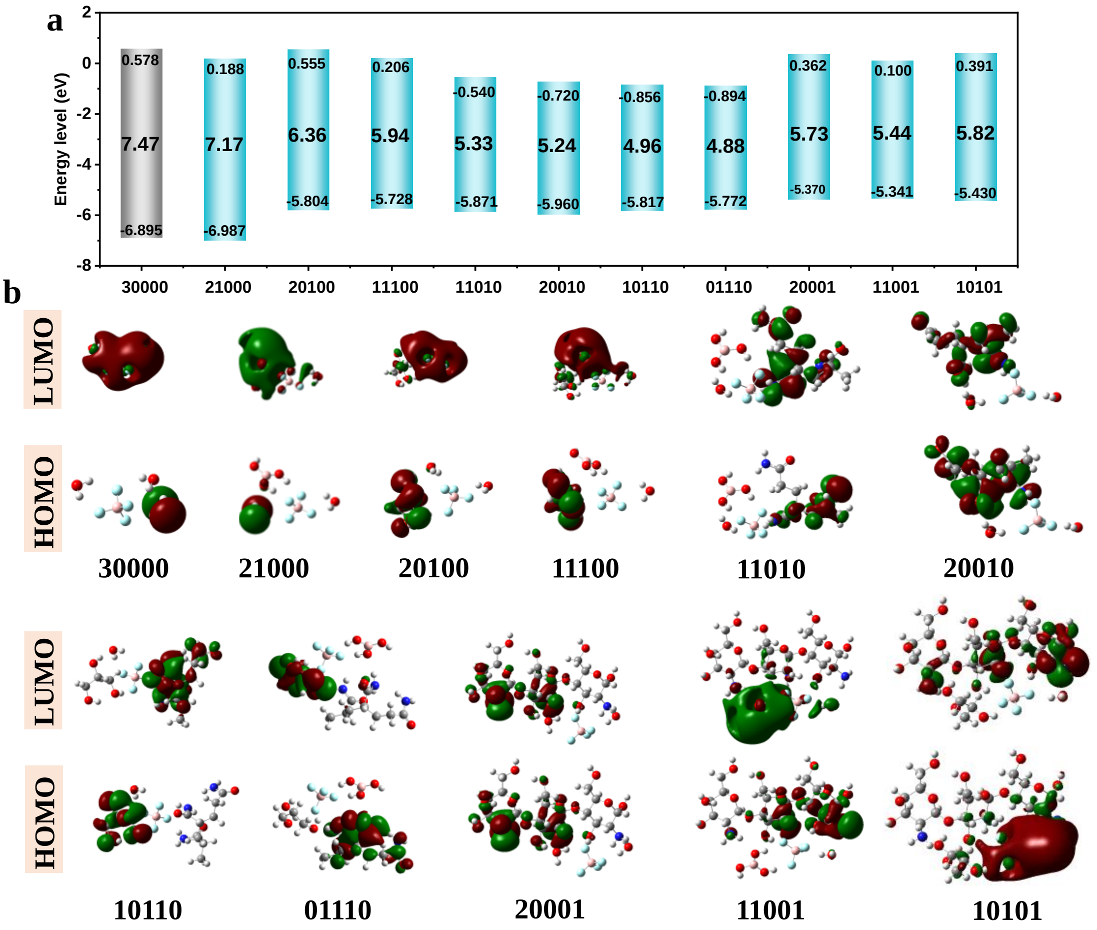

Supplementary Fig. 40 | a, Energy level and corresponding **b,** orbital structures of different solvation structures of free anions denoted as BF_4^- $(H_2O)_a(C_3H_8O_3)_b(H_3BO_3)_c(PAM)_d(CS)_e$ based on the B97-3c level DFT calculations.

For the 21000 mentioned in Supplementary Fig. 39, the electron states in the HOMO level are mainly occupied by the H_2O molecule. In contrast, the LUMO level is contributed from H_3BO_3 since the typical electron deficient characteristics in H_3BO_3 because of the *sp*2 hybridization. On the other hand, the LUMO level of $C_3H_8O_3$ is mainly contributed by anti-bonding orbitals with relatively low energy levels of -OH groups, whereas the HOMO level of $C_3H_8O_3$ is relatively higher due to the lone-pairs electrons in -OH groups. Thus, the solvation structures such as 10110 and 01110 consisting of $C_3H_8O_3$ exhibit lower energy gaps. As for the PAM-involved structures, the relatively higher HOMO can be attributed to the non-bonded electronic state of N and O in $-CONH_2$ groups from PAM. Similarly, the HOMO of CS is relatively higher

due to the more sufficient -NH_2 and -OH groups compared with PAM, which ensures the solvation structures involved with CS exhibit lower energy gaps. In conclusion, the mechanism of reduction of energy gaps of all the solvation structures tuned by the CRACSS strategy can be clarified from the prospective of electronic structures and energy levels.

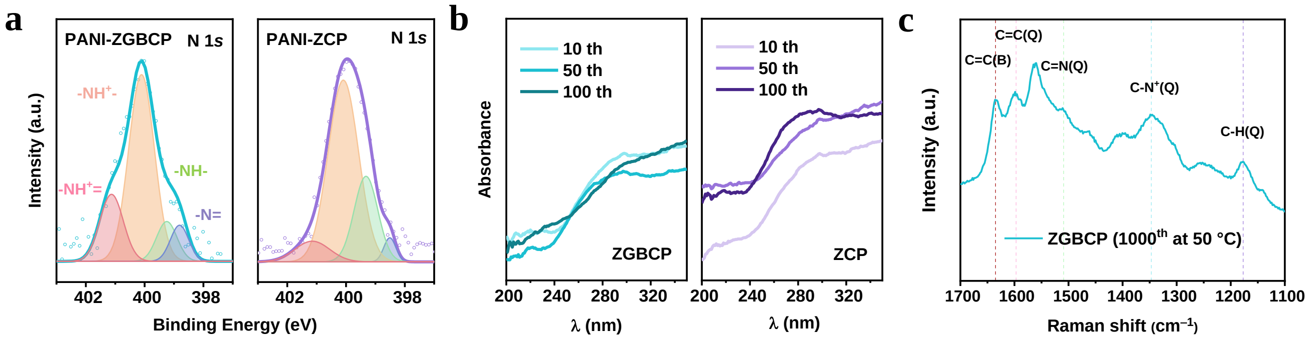

Supplementary Fig. 41 | Spectral characterization and mechanism analysis of PANI electrodes using ZGBCP and ZCP electrolyte. **a,** XPS N 1*s* spectra after 1000 cycles under room temperature. **b,** Evolution of the UV-Vis spectra along with the cycling under (50 °C). **c,** Raman spectra of the PANI cathode using ZGBCP electrolyte after 1000 cycles under 50 °C.

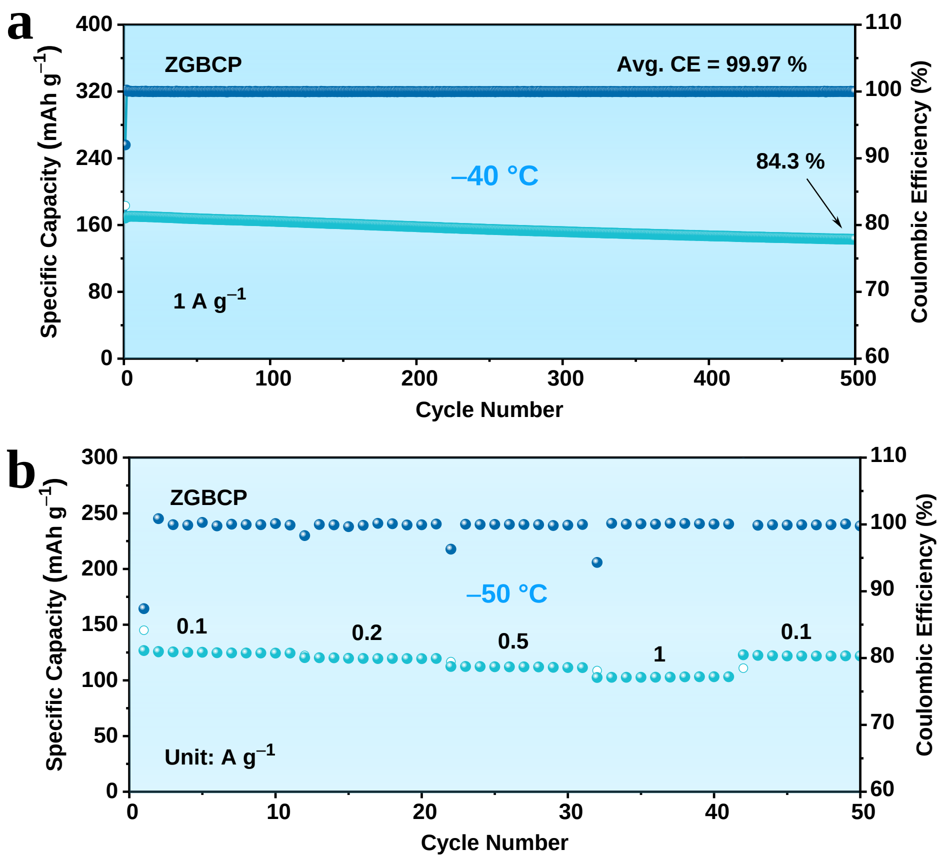

Supplementary Fig. 42 | Low temperature electrochemistry performance of Zn|ZGBCP|PANI full batteries. **a,** Cycling performance under -40 °C. **b,** Rate performance under -50 °C.

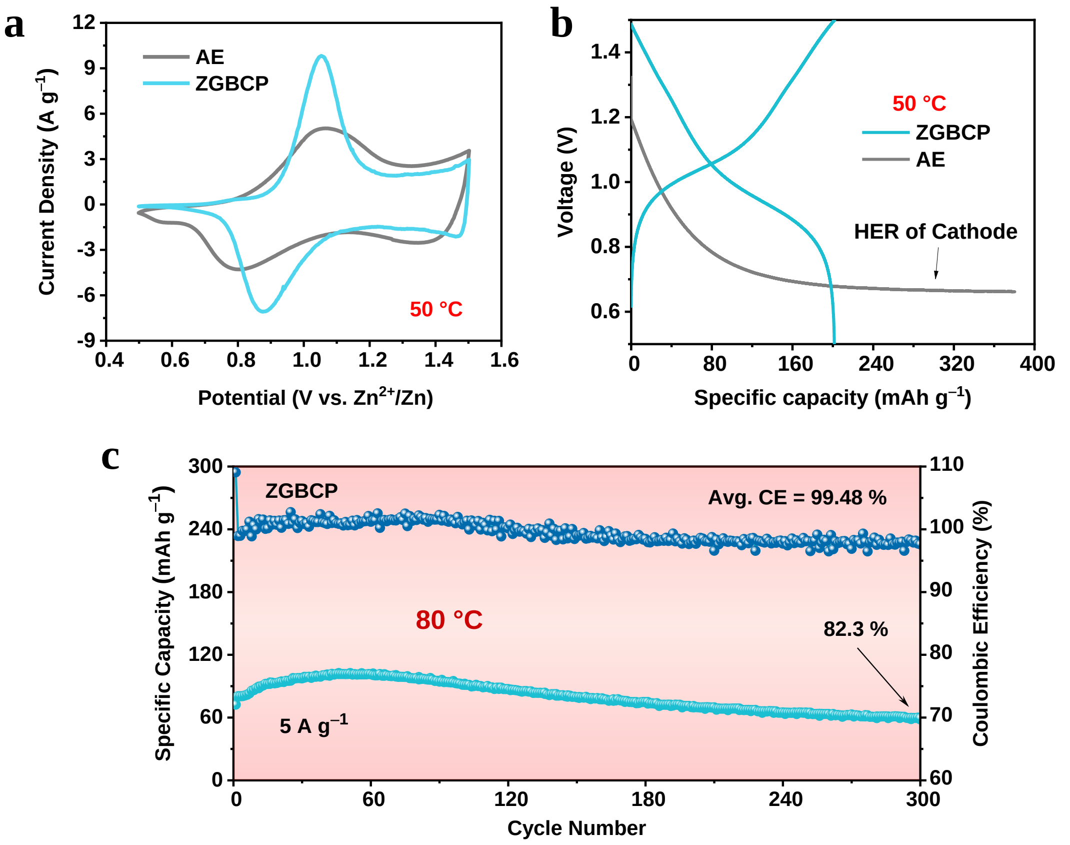

Supplementary Fig. 43 | Electrochemical performance of full batteries using AE and ZGBCP electrolytes under high temperatures. **a,** CV curves at 50 °C. **b,** GCD profiles at 50 °C. **c,** Cycling performance at 80 °C

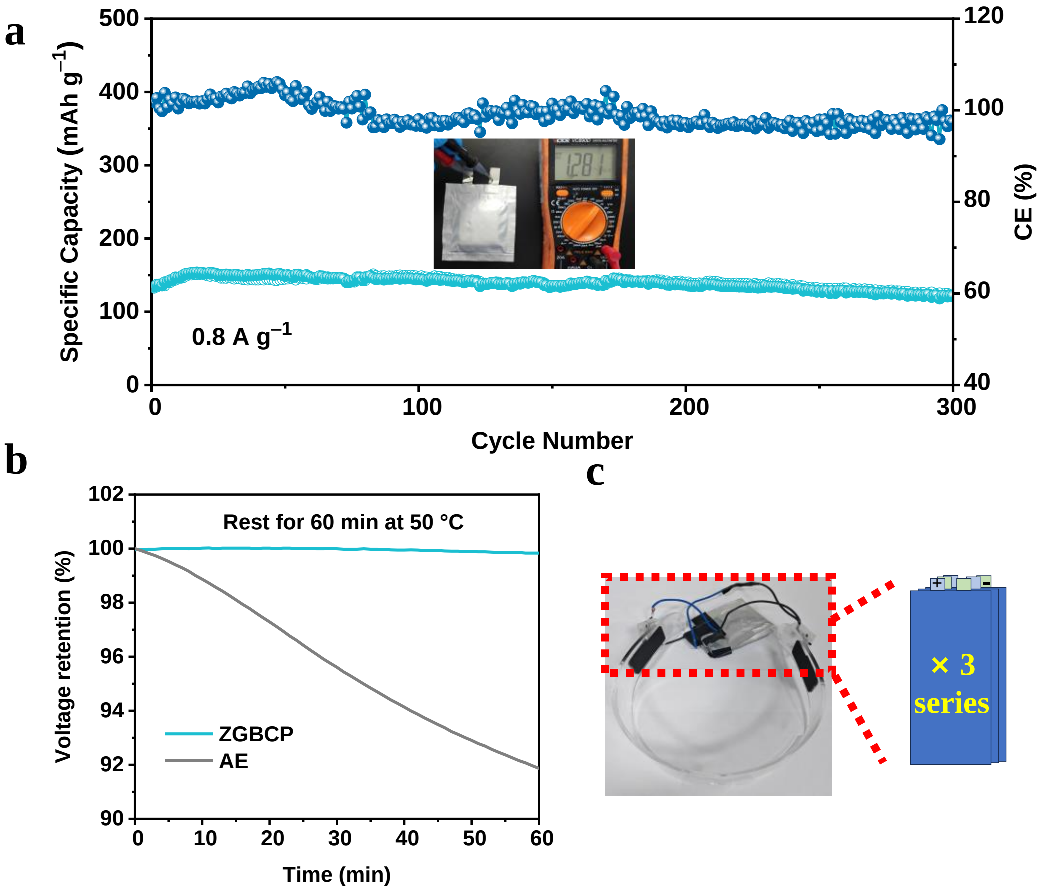

Supplementary Fig. 44 | The electrochemical performance and demonstration for powering electronic devices of Zn||PANI pouch-cells using ZGBCP electrolyte. **a,** Cycling performance. **b,** Voltage retention at 50 °C. **c,** Demonstration of wearable devices powered by the pouch-cell in series.

Table. 1 | The comparisons of solvation structures and performances of the reported electrolytes based on the $Zn(BF_4)_2$.

Ref	gel or liquid	Cationic solvation structure (representative)	Anionic solvation structure (representative)	Strategy	Overpotential under 1 mA cm^{-2} (mV)	Working temperature (°C)
[8]	liquid	$[Zn^{2+}(BF_4^-)_2(DME)(H_2O)_2]$	/	Organic Solvent	~170	+25
[9]	liquid	$[Zn^{2+}(BF_4^-)(H_2O)_2(ace)_3)]^+$	/	Organic Solvent	~80	0-+25
[10]	liquid	$[Zn^{2+}(BF_4^-)_2(H_2O)_4]^+$	/	Organic Solvent	/	-60-+25
[11]	liquid	$[Zn^{2+}(BF_4^-)_2(H_2O)_2(TMP)_2]^+$	/	Organic Solvent	200	+25
[6]	Gel	$[Zn^{2+}(BF_4^-)_2(H_2O)_2(PAM)]^+$	/	Saturated salts	/	-70-+25
**This work**	**Gel**	**$[Zn^{2+}(BF_4^-)(H_2O)_4(C_3H_8O_3)]^+$-PAM**	**$[BF_4^-(H_2O)(C_3H_8O_3)(H_3BO_3)]^-$**	**CRACCS**	**87**	**-50-+100**

Table 2. | Box length and molecule numbers of ZGBCP and AE system.

System	Box length (Angstrom)	H_2O	$Zn(BF_4)_2$	$C_3H_8O_3$	H_3BO_3	PAM	CS
ZGBCP_3mer	52	1520	118	128	57	55	1
ZGBCP_5mer	59	2550	197	213	95	55	1
AE	49	2861	206	\	\	\	\

Table 3. | The exchange current densities (mA/cm^2) of Zn stripping/plating reactions in different electrolytes containing varied amounts of boric acid (BA) and glycerol.

Boric acid **g,** / Glycerol **g,**	0.2	0.4	0.6	0.8
2	0.049	0.040	0.057	BA precipitation
4	0.045	0.043	0.032	BA precipitation
6	CS insoluble	CS insoluble	CS insoluble	BA precipitation

Supplemental References

1. Mendelovici E, et al. Cryogenic Raman spectroscopy of glycerol. *J. Raman Spectrosc.* **31**, 1121-1126 (2000).

2. Feng D, et al. Proton-Reservoir Hydrogel Electrolyte for Long-Term Cycling Zn/PANI Batteries in Wide Temperature Range. *Angew. Chem. Int. Ed.* **62**, e202215060 (2023).

3. Wan F, et al. An Aqueous Rechargeable Zinc-Organic Battery with Hybrid Mechanism. *Adv. Funct. Mater.* **28**, 1804975 (2018).

4. Liu Y, et al. Sulfonic-Group-Grafted $Ti_3C_2T_x$ MXene: A Silver Bullet to Settle the Instability of Polyaniline toward High-Performance Zn-Ion Batteries. *ACS Nano* **15**, 9065-9075 (2021).

5. Yan Y, et al. Tough Hydrogel Electrolytes for Anti-Freezing Zinc-Ion Batteries. *Adv. Mater.* **35**, 2211673 (2023).

6. Shi Y, et al. An Anti-Freezing Hydrogel Electrolyte for Flexible Zinc-Ion Batteries Operating at −70 °C. *Adv. Funct. Mater.* **33**, 2214546 (2023).

7. Li X, et al. Flexible Wide-Temperature Zinc-Ion Battery Enabled by an Ethylene Glycol-Based Organohydrogel Electrolyte. *ACS Appl. Energy Mater.* **4**, 12718-12727 (2021).

8. Meng C, et al. A eutectic electrolyte for an ultralong-lived Zn//V_2O_5 cell: an in situ generated gradient solid-electrolyte interphase. *Energy Environ. Sci.* **16**, 3587-3599 (2023).

9. Wang G, et al. Gradient-Structured and Robust Solid Electrolyte Interphase In Situ Formed by Hydrated Eutectic Electrolytes for High-Performance Zinc Metal Batteries. *Adv. Energy Mater.* **14**, 2303549 (2024).

10. Wang D, et al. Localized Anion-Cation Aggregated Aqueous Electrolytes with Accelerated Kinetics for Low-Temperature Zinc Metal Batteries. *Angew. Chem. Int. Ed.* **62**, e202315834 (2023).

11. Ma G, et al. Zn metal anodes stabilized by an intrinsically safe, dilute, and hydrous organic electrolyte. *Energy Storage Mater.* **54**, 276-283 (2023).
